# Microplastics of Broad Size Range Reduce Bacteriophage Activity in Aqueous Environments

**DOI:** 10.1021/acs.jpcb.5c01316

**Published:** 2025-06-10

**Authors:** Enkhlin Ochirbat, Rafał Zbonikowski, Michał Folga, Magdalena Bonarowska, Jan Paczesny

**Affiliations:** † Institute of Physical Chemistry, 49559 Polish Academy of Sciences, Kasprzaka 44/52, 01-224 Warsaw, Poland; ‡ Faculty of Chemistry, Warsaw University of Technology, Noakowskiego 3, 00-664 Warsaw, Poland

## Abstract

Microplastics, pervasive environmental contaminants, attract significant attention due to their detrimental effects across ecosystems. Reports show the presence of microplastics in water, soil, aqueous organisms, and even human tissues and blood. This study investigates the impact of microplastics on bacteriophages, i.e., viruses that play crucial roles in regulating microbial communities and maintaining ecological balance. Since bacteriophages lyse up to 40% of bacterial populations daily, their role in environmental stability is paramount. We demonstrate that microplastics can reduce the apparent number of active bacteria in aquatic environments. To explore the interaction between microplastics and bacteriophages, we examine the effects of various microplastic types (polystyrene, poly­(vinyl chloride), polyethylene, and polyethylene terephthalate) and size ranges of particles on phages of varying morphologies (tailed T4, filamentous M13, and icosahedral MS2). Additionally, we assess the influence of bacterial debris, representing organic matter, on the heteroaggregation of microplastic particles and phages. Our findings reveal a significant decline of up to 99.99% in active phages, underscoring the profound effects of microplastics on phage dynamics. These results provide critical insights into the complex interactions between microplastics and phages, highlighting the need for urgent action to address microplastic pollution.

## Graphical abstract

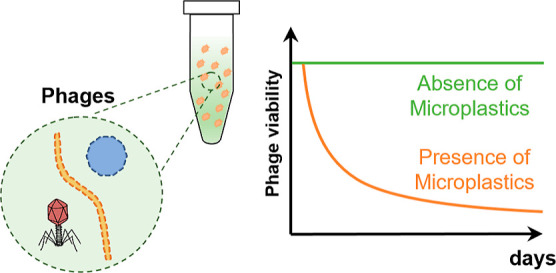

## Introduction

Plastics are integral to modern life, with global production exceeding 400 million tons annually and projected to double within the next decade.^1^ Plastics are synthetic organic polymers characterized by high-molecular-weight molecules with elongated backbone chains. They are primarily derived from hydrocarbons sourced from fossil fuels.^2^ During manufacturing, various additives are incorporated to enhance the properties of these polymers, imparting strength, durability, lightweight characteristics, and resistance to corrosion.^3,4^ A few types of plastics dominate the market, accounting for 75% of the total demand: polyethylene (PE), polyethylene terephthalate (PET), polystyrene (PS), polyvinyl chloride (PVC), polypropylene (PP), and polyurethane (PU).^5^

However, plastic waste poses a severe environmental threat. Over 8 million tons of plastic are dumped annually into oceans, and less than 15% of the total waste is recycled.^6,7^ The environmental half-lives of plastics vary significantly, ranging from days to thousands of years, depending on the polymer type and environmental conditions.^8^ Fragmentation processes, caused, for example, by exposure to UV radiation and mechanical forces, lead to the formation of microplastics—plastic particles smaller than 5 mm.^9^ Microplastics are classified as either secondary (formed by fragmentation) or primary (intentionally manufactured for specific applications, such as microbeads in personal care products and industrial abrasives).^10^

Microplastics are widespread in marine environments, driven by hydrodynamic processes, wind, and ocean currents. The Great Pacific Garbage Patch alone contains an estimated 1.69 trillion microplastic fragments, making up 94% of its floating debris.^11^ Other studies estimate around 5 trillion microplastic particles, weighing approximately 250,000 tons, in the world’s oceans.^12^ Various marine organisms, from plankton to large mammals, ingest these particles, leading to physical damage, malnutrition, and reduced feeding efficiency.^13,14^ Microplastics act as vectors for pollutants like heavy metals and toxic chemicals, which organisms can absorb.^15,16^ The particles disrupt the development and function of marine organisms and affect entire ecosystems by altering community structures and interspecies interactions.^17,18^ Additionally, microplastics can influence the microbiome in water, affecting processes like organic matter decomposition and nitrogen fixation.^19^ Besides aquatic environments, microplastic is also found in soil, plants, human tissues, and blood.^20–22^ Micro- and nanoplastics can adsorb biologically active compounds, such as antibiotics, altering their stability, bioavailability, and biological effects, potentially contributing to antibiotic resistance and impacting microbial communities.^23^

Our recent research highlights that microplastics can also impact entities at the very base of the food chain: bacteriophages.^24^ Bacteriophages, or phages, are pivotal in regulating bacterial populations, causing daily reductions of 15–40% in oceans, 10–50% in surface waters, and up to 100% in harsh environments.^25,26^ By controlling bacterial populations, phages significantly influence ecosystem nutrient cycling and energy flow. Despite growing interest in the separate effects of microplastics and bacteriophages, their potential interactions remain poorly understood.

Our earlier publication explored the heteroaggregation mechanisms between virions and microplastics, focusing primarily on adsorption-driven phage scavenging and the secondary impact of leachables from various microplastic types.^24^ This study investigates the interactions between bacteriophages and microplastics in aqueous environments, in both the presence and absence of organic matter derived from sonicated bacterial debris. We selected polyethylene terephthalate (PET), polystyrene (PS), polyvinyl chloride (PVC), and polyethylene (PE) due to their high prevalence in environmental waste and their potential impact on phage activity.^24^ Microplastics were categorized into four size ranges (<100 μm, 100–500 μm, 500–1000 μm, and 1000–5000 μm) to assess whether particle size influences phage inactivation. Phage concentrations were monitored at three time points: 1 h, 24 h, and 7 days, to capture immediate and prolonged effects. By clarifying these interactions, this research provides critical insights into the implications of microplastic pollution, potentially informing strategies to mitigate its impact on microbial communities and ecosystem dynamics.

## Materials and Methods

### Chemicals

LB-agar contained 15 g/L agar, 10 g/L NaCl, 10 g/L tryptone, and 5 g/L yeast extract and was used as an instant mix (Carl Roth, Germany). LB-medium had the same composition except for the lack of 15 g/L agar (Carl Roth, Germany). TM buffer (pH = 7.4) was prepared using 10 mM Tris base, 5 μM CaCl_2_, 10 mM MgSO_4_, and Milli-Q water. PBS buffer (pH = 7.4) was prepared by dissolving 1 PBS tablet (MP Biomedicals, USA) in 100 mL of Milli-Q water. All chemicals were purchased from Sigma-Aldrich (Germany). All solutions were sterilized by autoclaving (3150EL, Tuttnauer, Germany) before use.

### Preparation of the Bacteriophages

Bacteriophages MS2, T4, and M13 were chosen due to their distinct morphologies (icosahedral, tailed, and filamentous, respectively). These types represent the majority of phages, with tailed phages being the most prevalent. Lytic phages were used for this study.

Phages were obtained from Phage Consultants, Poland. An early logarithmic culture of Escherichia coli BL21 was infected by T4. For MS2 and M13 multiplication, the E. coli C3000 strain was used. After lysis, T4 and M13 phages were precipitated using polyethylene glycol. The T4 and M13 precipitates were purified by centrifugation and diluted with 1 M NaCl. Then, CsCl gradient centrifugation was performed (Beckman Optima XL70 ultracentrifuge, Ti50 rotor, 100,000 *g*), following the method described by Sambrook et al.^27^ A step gradient (ρ = 1.6, 1.5, 1.3 g/mL) was prepared in ultraclear tubes, and 4 mL of concentrated phage suspension was layered on top. After centrifugation at 100,000 *g* for 4 h at 4 °C, the phage band was visually located, extracted with a 26-gauge needle, and collected for dialysis. T4 and M13 were dialyzed using 12–14 kDa regenerated-cellulose tubing, prehydrated in TM buffer for 24 h. The loaded and sealed tubing was placed in TM buffer on a magnetic stirrer in a refrigerated setting, with daily buffer changes. Dialysis continued until the solution became transparent, after which the phage suspension was collected and stored at 4 °C in the dark with minimal headspace. Afterward, 0.2 μg/mL viscolase (A&A Biotechnology) was added to samples with phages T4 and M13 to digest the residual DNA remaining in the TM buffer after the procedure. In the case of MS2, the lysate was only filtered using 0.22 μm syringe filters. The initial concentration of the T4 phage stock was 2 × 10^13^ PFU/mL, determined by plaque assay on E. coli BL21 bacteria. In case of MS2 and M13, the initial concentrations were 1.5 × 10^11^ PFU/mL and 1.2 × 10^10^ PFU/mL, respectively. For the experiments, we chose 10^5^ PFU/mL phages.

### Preparation of Medium with Sonicated Bacterial Debris (Biomedium)

To simulate the presence of organic debris in aquatic environments, a medium was prepared using Staphylococcus aureus bacteria, which was chosen because it cannot be infected by any of the phages selected for this study. The bacterial culture was initiated by growing S. aureus overnight in LB medium at 37 °C (ES-20, Biosan, Latvia). After 24 h, the culture was refreshed with fresh LB medium until the OD_600_ reached 0.4, indicating a bacterial concentration of 10^7^ to 10^8^ CFU/mL. The culture was diluted to a final concentration of 5 × 10^4^ CFU/mL in TM buffer. The chosen bacterial cell concentration falls within the typical range of marine bacteria (5 × 10^4^–10^6^ cells/mL) observed in ocean waters, depending on the depth and environmental conditions.^28^ To disrupt the bacterial cells, the suspension was subjected to ultrasonication using an ultrasonic homogenizer (VCX 130, Sonics & Materials, USA) for a total duration of 1 h and 15 min, with intermittent 10 s pulses. The test tube containing the bacterial suspension was placed on dry ice throughout the process to prevent overheating caused by the ultrasonic homogenizer. We termed this mixture biomedium (BM). The effectiveness of the cell disruption was confirmed using the plating method, which verified the absence of any viable bacterial cells.

### Preparation of Microplastic

Four types of plastics were selected to prepare the microplastic samples: polystyrene (PS), poly­(vinyl chloride) (PVC), polyethylene terephthalate (PET), and polyethylene (PE) (Figure 1A). All polymer materials were obtained from a local supplier of commercial polymer products. These materials are representative of common environmental microplastic sources.^24^ Polymer pieces, each a few cubic centimeters in size, were cleaned using ethanol-soaked paper towels and rinsed thoroughly with ultrapure water. The outer surface layer of each polymer piece was removed with a sharp scalpel to expose fresh material, which was then mechanically scraped off using either a scalpel or a Dremel multigrinder. The resulting fine particles were collected in glass containers, rinsed with ultrapure water, and dried.

**Figure 1:**
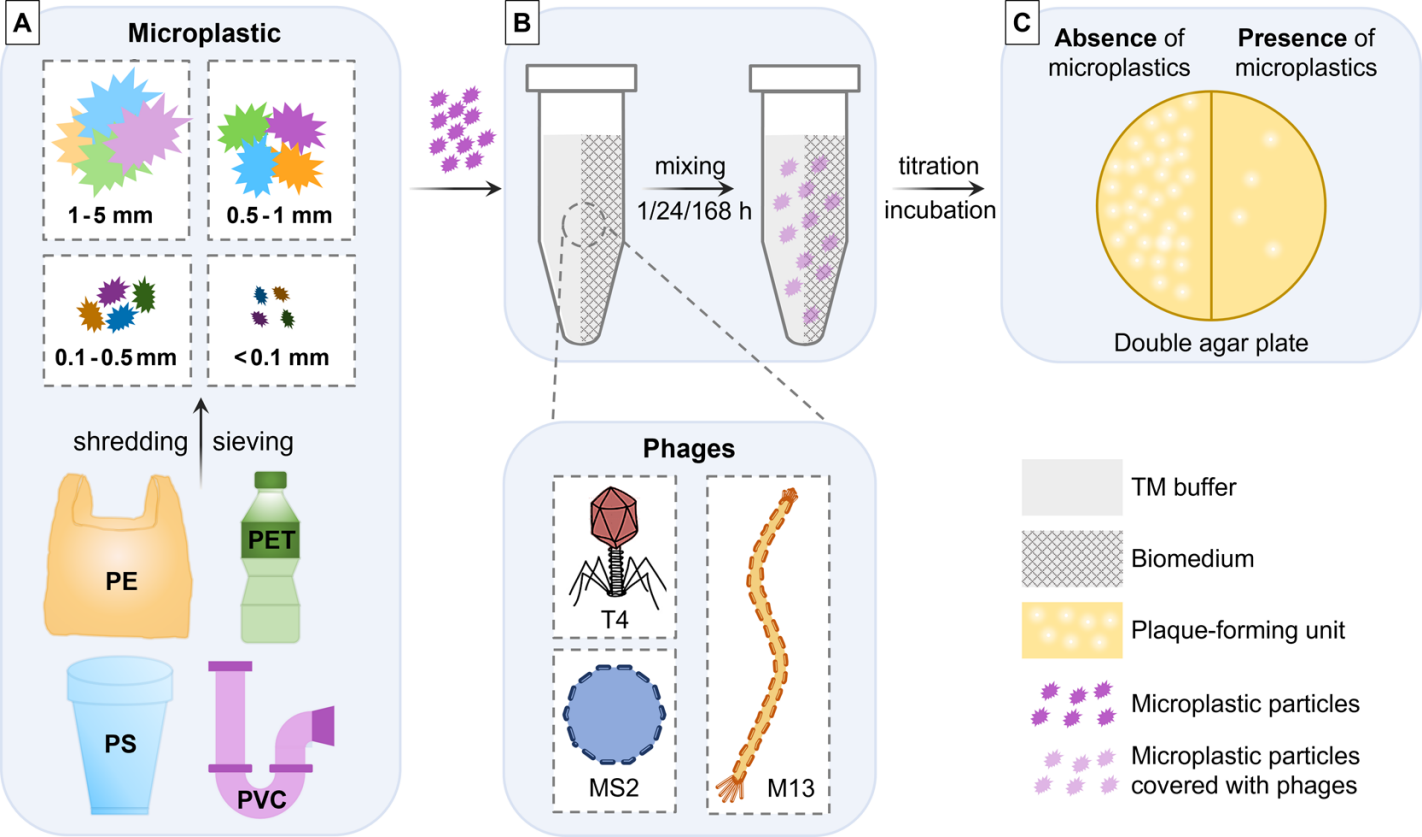
Schematic representation of the experimental workflow for investigating the effect of microplastics on bacteriophage viability. (A) Microplastic preparation and size classification. Four widely used plastic types, polyethylene (PE), polyethylene terephthalate (PET), polystyrene (PS), and polyvinyl chloride (PVC), were processed into microplastic particles by shredding and sieving, yielding four distinct size fractions: 1–5 mm, 0.5–1 mm, 0.1–0.5 mm, and <0.1 mm. (B) Phage-microplastic incubation. Each bacteriophage, T4 (tailed, dsDNA), M13 (filamentous, ssDNA), and MS2 (icosahedral, ssRNA), was separately incubated with each microplastic type and size fraction in either TM buffer or biomedium for 1, 24, or 168 h to assess adsorption and potential inactivation. (C) Plaque assay for phage viability. Following incubation, samples were titrated and plated using the double agar overlay method to quantify infectious phages. A reduction in plaque-forming units (PFUs) in the presence of microplastics indicates potential phage adsorption onto microplastic surfaces.

Three stainless steel sieves (Polmarkus, Poland) with different mesh diameters were employed to classify the microplastic particles by size: 5 mm, 1 mm, and 0.5 mm. The sieves were stacked in descending order of mesh size, and the microplastic samples were placed on top. The samples were agitated using a shaker operating at 300 rpm, effectively separating the particles into size fractions. The first two fractions were collected directly from the sieves. Particles smaller than 500 μm were further refined using a polyester mesh sieve (Gilson, USA) with a 100 μm mesh size. The samples were shaken again to separate finer particles, yielding two additional size fractions. The microplastic particles were then categorized into four distinct size fractions: (1) 1000–5000 μm, (2) 500–1000 μm, (3) 100–500 μm, and (4) <100 μm. This comprehensive size categorization allowed for a detailed analysis of the microplastics, facilitating a more nuanced examination of their interactions with bacteriophages in subsequent experiments.

### Preparation of Bacteriophages with Microplastic Sample

A 1 mg sample of microplastic particles was weighed and transferred from each size group into 1.5 mL Eppendorf tubes. The samples were then divided into two experimental conditions: (1) bacteriophage suspension in TM buffer and (2) bacteriophage suspension in biomedium (containing debris of nonhost bacteria). For each microplastic size group, three Eppendorf tubes were prepared for both the TM buffer and the biomedium, maintaining triplicate samples for statistical analysis. Next, 1 mL of the corresponding bacteriophage suspension was added to each tube, resulting in a consistent microplastic concentration of 1 mg/mL (Figure 1B). Samples containing only TM buffer and the phage suspension, but without microplastic, were prepared as a control. Separate control samples with biomedium but without microplastics were included to evaluate the influence of organic matter on phage infectivity. For all experiments, phage suspensions were adjusted to a final concentration of 1 × 10^5^ PFU/mL. The tubes were mixed at a speed of 80–90 rpm to ensure uniform distribution of the microplastics and phages. Titration of bacteriophages was performed at three time points: 1 h, 24 h, and 7 days postincubation.

### Evaluation of the Number of Active Phages in the Suspension—Double Agar Overlay Assay

A double overlay method and droplet plaque counting test were conducted to assess phage activity and virulence (Figure 1C). A 4 mL portion of 0.5% agar in LB medium was first cooled down to 56 °C and then mixed with 200 μL of refreshed bacteria (i.e., overnight culture diluted in fresh LB medium and incubated for 2 h to reach OD_600_ = 0.4). E. coli BL21 (T4 phage) or E. coli C3000 (MS2 and M13 phages) were cultured depending on the phage type. The solution was poured onto a previously prepared Petri dish with LB-agar (LB medium and 1.5% agar). After the agar with the bacteria solidified, at least eight technical replicates (5 μL of each droplet) of adequately diluted phage suspension were deposited onto the plate. Subsequently, the plates were incubated at 37 °C (BD3, Binder, Germany) for 24 h. After removing the plates from the incubator, the plaques were counted, and the concentration of phages was calculated and expressed in PFU/mL (plaque-forming units). The experiments were performed in three replicates. To evaluate the impact of microplastics on phage activity, a Student’s *t*-test was performed, with significance levels indicated as follows: *p < 0.05, **p < 0.01, and ***p < 0.001.

### Brunauer, Emmett, and Teller Measurement

Surface areas, total pore volumes, and average pore size of polymers were determined by a Micromeritics ASAP 2020 analyzer. The weight of the samples was ∼0.3 g. Before measurements, the samples were degassed in a vacuum at 343 K for 5 h to clean their surface.

The adsorption process was carried out at liquid nitrogen temperature (77 K), and krypton was used as the adsorbent instead of the commonly used nitrogen. For samples with an extremely low specific surface area (and these are the polymers discussed in this article), using krypton significantly improves the accuracy of the measurements. Specific surface area calculations were carried out according to the Brunauer–Emmet–Teller (BET) theory. The BET model was applied in the range of p/p0 from 0.05 to 0.30, and the BET plots obtained showed linearity (the measurement error level was 5%). The Horwath-Kawazoe method was used to determine the volume and average pore width. Some samples contained micropores with dimensions of less than 2 nm; the surface area of these micropores was determined using the t-plot method.

### Dynamic Light Scattering and Zeta-Potential Measurements

DLS and ζ-potential measurements were carried out using a Zetasizer Nano ZS apparatus (Malvern Instruments Ltd., Malvern, U.K.) equipped with a DLS module (He–Ne laser 633 nm, max 4 mW, allowing for measuring backscattered (173°) and forward-scattered (12.8°) light). Quasi-backscattered light was used for the measurements.

### Multivariate Analysis (Ordinary Least Squares Method)

Linear regressions and diagnostic tests were conducted with the Python script, which is provided in the Supporting Information (Section S2.1.).

## Results

### Characterization of Microplastics and Bacteriophages

BET analysis of microplastics revealed the increase in surface area per milligram of the polymers from fractions 2 to 4, i.e., with the decreasing size of particles. This was expected. An opposing trend was observed in the case of BET sizes, as expected. For fraction 4, this parameter ranged from around 25 to 50 μm. Fractions 1 and 2 were similar, as both were rather large, and the BET parameters for these two fractions did not follow the trends. This was most likely due to the sample preparation process. The exact results of the BET analysis are given in the Supporting Information (Table S1).

A decrease in size was observed with decreasing sieve size (Figure 2). However, the polydispersity of the samples was quite large, and we did not attempt to average the measured sizes. We followed BET sizes, which were averaged over a large number of particles.

**Figure 2:**
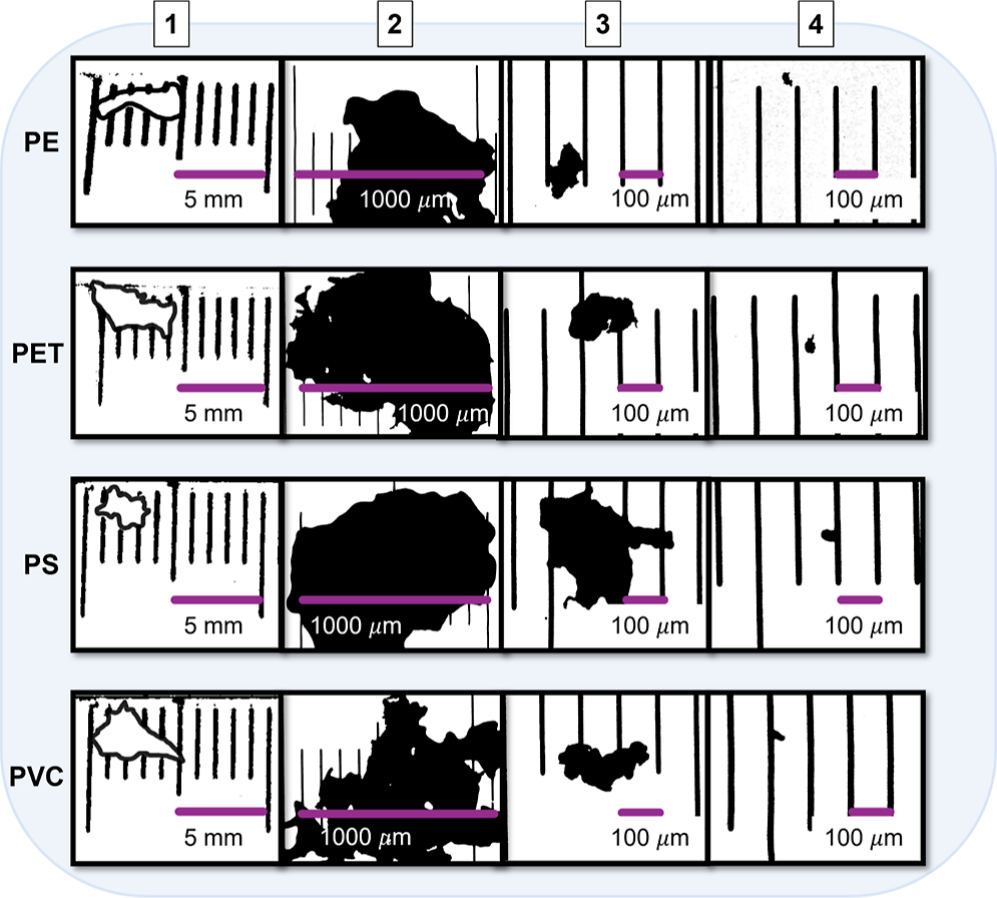
Size measurement of microplastics using a precision stage micrometer. The figure presents exemplary microscopic pictures of polyethylene (PE), polyethylene terephthalate (PET), polystyrene (PS), and polyvinyl chloride (PVC) microplastics with a precision stage micrometer following the sieving process. The columns labeled 1 to 4 correspond to progressively smaller microplastic size fractions: (1) 1–5 mm, (2) 500–1000 μm, (3) 100–500 μm, and (4) < 100 μm.

DLS and ζ-potential measurements were performed to determine the hydrodynamic radius and surface charge of the T4, M13, and MS2 bacteriophages in the TM buffer. The results are summarized in Table 1. The hydrodynamic radii of M13, MS2, and T4 phages were measured as 37.0, 22.3, and 66.7 nm, respectively. Zeta potential analysis revealed negative surface charges for all phages, with M13 at −13.0 mV, MS2 at −14.5 mV, and T4 at −21.6 mV. The obtained values were in line with the available literature.^29–32^ We hypothesize that the differences in phage size and electrostatic properties may influence their interactions with microplastics.

**Table 1:** Average Results of Hydrodynamic Radius and Zeta Potential for M13, MS2, and T4 in TM Buffer

bacteriophage	hydrodynamic radius [nm]	std bev. [nm]	ζ-potential [mV]	std dev. [mV]
M13	37.0	20.7	–13.0	7.4
MS2	22.3	7.6	–14.5	6.7
T4	66.7	14.8	–21.6	6.3

### Effect of Microplastics on Bacteriophages

In this section, we examined the impact of four types of microplastics, polyethylene (PE), polyethylene terephthalate (PET), polystyrene (PS), and polyvinyl chloride (PVC) at concentrations of 1 mg/mL, on the activity of three distinct bacteriophages (T4, M13, MS2). The bacteriophages were exposed to microplastic particles of varying sizes (1–5 mm, 0.5–1 mm, 0.1–0.5 mm, and <0.1 mm) in both TM buffer (TM) and biomedium (BM) over three time points (1, 24, and 168 h). The phage activity was measured using titration and is presented in Figures 3, 4, and 5. The exact values are given in Tables S2–S4. Graphs illustrating the activity of each phage in TM and BM conditions, presented both separately and together, across all microplastic size fractions, are provided in the Supporting Information (Figures S1–S13). Control samples labeled “TM” and “BM” represent phage suspensions in TM buffer and biomedium, respectively, without microplastics. Statistical comparisons are marked with asterisks: black asterisks indicate significant differences between each microplastic condition and its respective control, while colored asterisks indicate differences between TM and BM conditions for the same microplastic size fraction.

**Figure 3:**
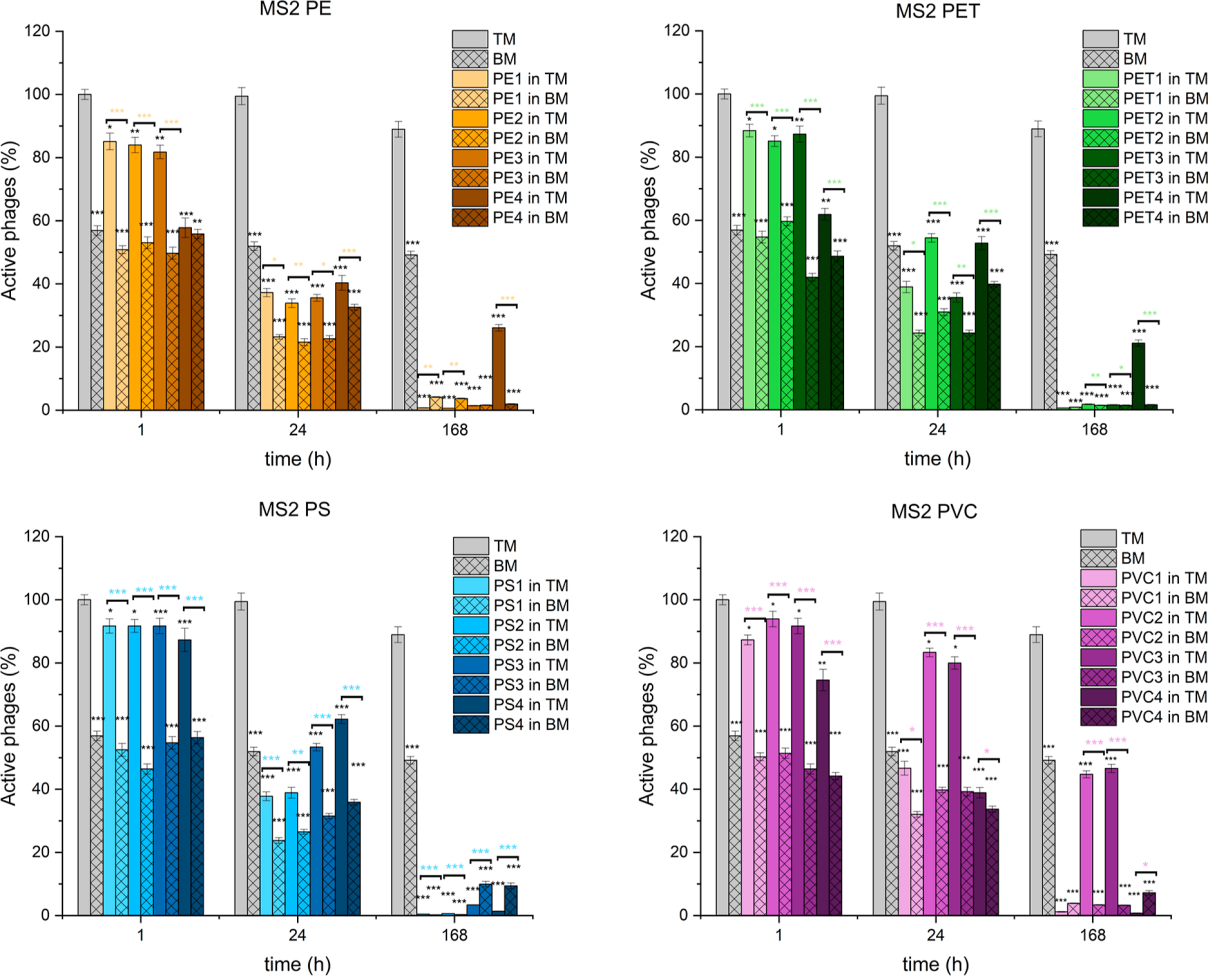
Effect of different microplastics on MS2 phage activity over time. The percentage of active MS2 phages is presented when exposed to four types of microplastics across 1, 24, and 168 h in two media conditions: TM buffer (bars without pattern) and biomedium containing sonicated bacterial debris (bars with pattern). Phage activity is expressed as a percentage of the initial titer, with mean values shown and error bars representing the standard deviation.

**Figure 4:**
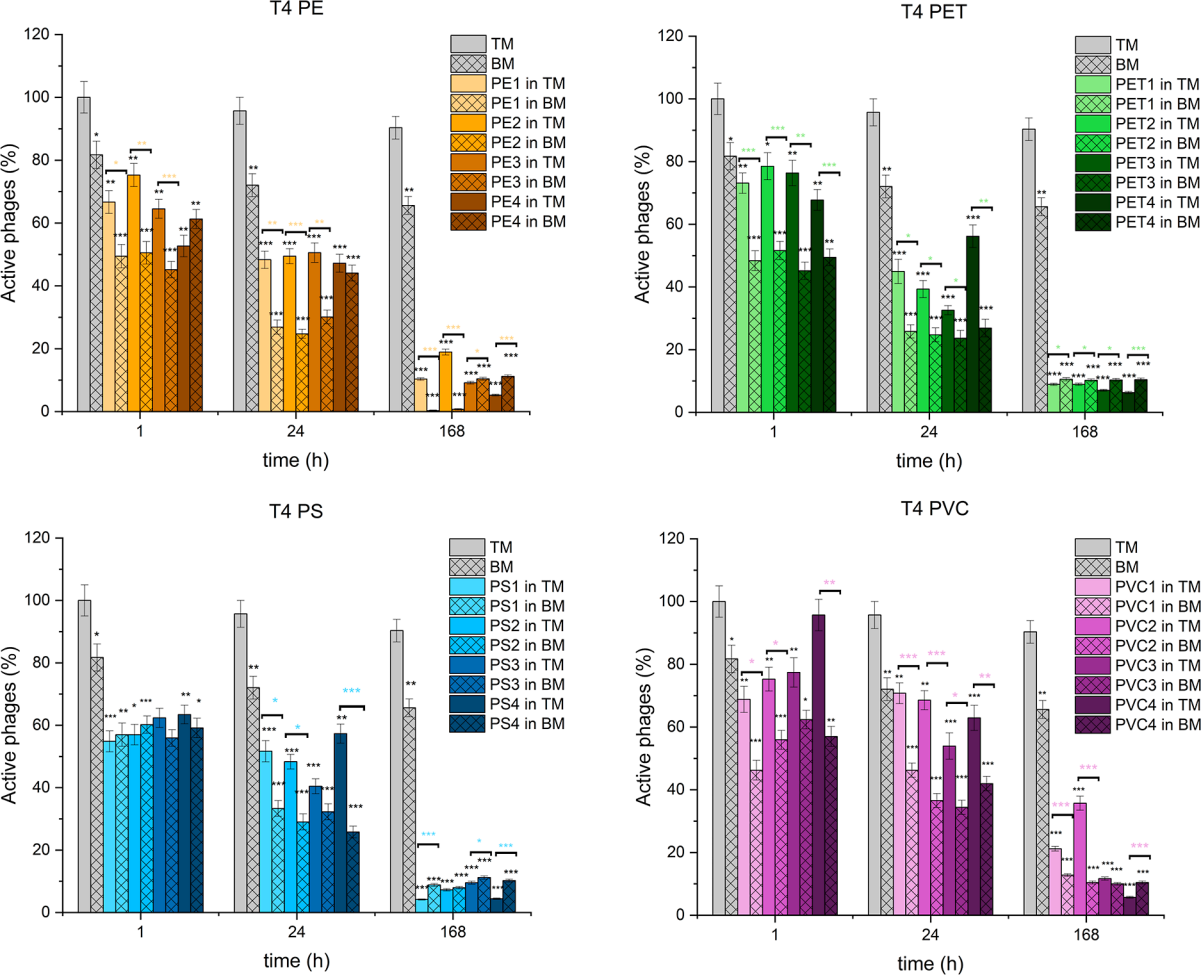
Effect of different microplastics on T4 phage activity over time. The percentage of active T4 phages is presented when exposed to four types of microplastics across 1, 24, and 168 h in two media conditions: TM buffer (bars without pattern) and biomedium containing sonicated bacterial debris (bars with pattern). Phage activity is expressed as a percentage of the initial titer, with mean values shown and error bars representing the standard deviation.

**Figure 5:**
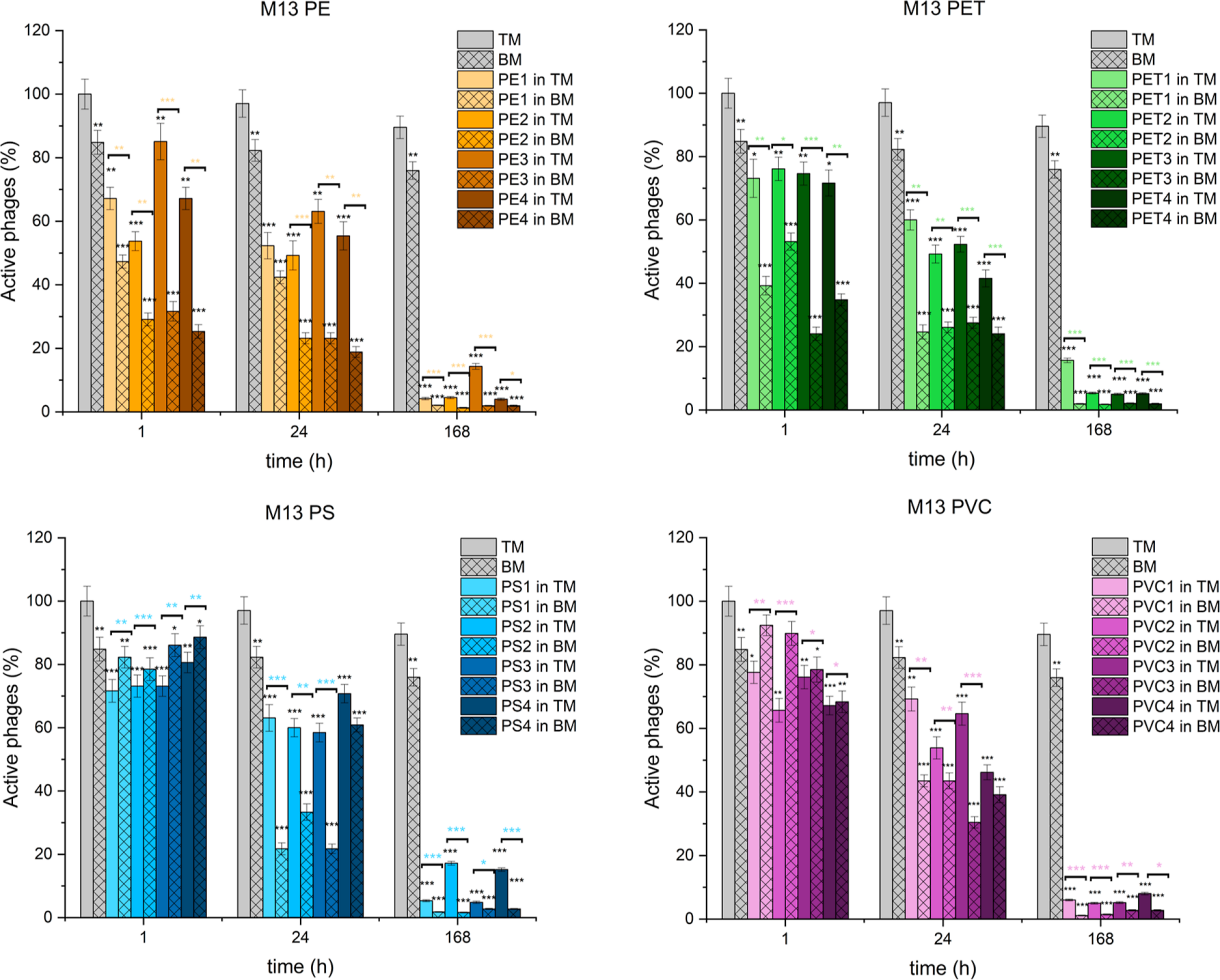
Effect of different microplastics on M13 phage activity over time. The percentage of active T4 phages is presented when exposed to four types of microplastics across 1, 24, and 168 h in two media conditions: TM buffer (bars without pattern) and biomedium containing sonicated bacterial debris (bars with pattern). Phage activity is expressed as a percentage of the initial titer, with mean values shown and error bars representing the standard deviation.

### Effect of Microplastics on MS2 Phage

In the presence of PE microplastics, MS2 phage activity decreased by 15–20% for size fractions PE1, PE2, and PE3 (100–5000 μm) after 1 h of incubation in TM, while a larger reduction of 40% was observed for PE4 (<100 μm). The reduction was more pronounced in BM, with a 40–50% decrease across all size fractions. After 24 h, phage inactivation increased to 60–65% for all PE sizes in TM and 65–80% in BM. By 168 h, nearly all phages were inactive, except for the smallest size fraction (PE4) in TM, which showed only a 70% reduction.

For PET microplastics, a moderate reduction in MS2 phage activity was observed after 1 h, with a 10% decrease for PET1, PET2, and PET3 and a 40% reduction for PET4. In BM, phage inactivation ranged from 40% to 60% across all sizes. At the 24 h mark, a greater reduction was noted, particularly for PET1 and PET3, with phage activity decreasing by 45–65% in TM and 60–75% in BM. After 168 h, nearly complete inactivation was observed for all PET sizes except for PET4, which retained about 20% activity.

MS2 phage activity showed a 10% decrease in TM and a 40–50% decrease in BM after 1 h of exposure to PS microplastics. At 24 h, phage inactivation was higher, with 40–60% reductions in TM and 65–75% in BM. The greatest inactivation was seen for size fractions PS1 and PS2 (500–5000 μm). By 168 h, almost complete inactivation was observed across all conditions, except for PS3 and PS4 in the biomedium, where a 90% reduction was noted.

Exposure to PVC microplastics resulted in significant reductions in MS2 phage activity, particularly for size fractions PVC1 (1000–5000 μm) and PVC4 (<100 μm). After 1 h, a 5–25% decrease in phage titer was noted in TM, while BM samples showed a 50–55% reduction across all sizes. At 24 h, phage inactivation was more pronounced for PVC1 and PVC4, with 50–60% decreases in TM buffer. In contrast, PVC2 and PVC3 showed a smaller 15–20% reduction. In BM, phage inactivation reached 60–70% across all size fractions. After 168 h, nearly total inactivation was observed for PVC1 and PVC4 in TM, while PVC2 and PVC3 showed a 50–55% reduction. In BM, more than 90% of the phages were inactivated for all PVC sizes.

### Effect of Microplastics on T4 Phage

After 1 h of incubation in TM buffer, T4 phage activity decreased by 25–35% for PE1, PE2, and PE3 (100–5000 μm), while PE4 (<100 μm) showed a stronger reduction of nearly 50%. The reduction was more pronounced in BM, with a 40–55% decrease across all size fractions. At 24 h, phage inactivation increased to 50–55% in TM buffer and 55–75% in BM across all size fractions. By 168 h, 85–95% of the phages were inactive in the TM buffer, while in BM, phage viability dropped by 90% to almost complete inactivation (100%).

In the presence of PET, a moderate reduction in T4 phage activity was observed after 1 h, with a 20–30% decrease in TM buffer and a 50–55% decrease in BM across all size fractions. At 24 h, phage activity further declined, showing a 45–70% reduction in TM buffer and approximately 75% in BM. By 168 h, the effect was comparable across all PET sizes, leading to 90–95% inactivation in both TM buffer and BM.

T4 phage activity exhibited a 35–45% decrease in both TM buffer and BM across all PS size fractions after 1 h. At 24 h, a more pronounced effect was observed, with phage activity declining by 45–60% in TM buffer and 65–75% in BM. By 168 h, the number of active phages had dropped by 80–95% in both environments, with slight variation between microplastic sizes.

After 1 h, T4 phage activity was reduced by 25–40% for PVC1, PVC2, and PVC3 (100–5000 μm), while PVC4 (<100 μm) showed only a minor reduction (∼5%) in TM. In BM, the reduction was 35–45% for PVC2, PVC3, and PVC4 (<1000 μm) but significantly higher (∼75%) for PVC1 (1000–5000 μm). At 24 h, phage inactivation increased to 35–50% in TM and 55–65% in BM across all size fractions. By 168 h, 70–95% of phages were inactivated in TM, while in BM, phage viability dropped by 85–90% across all PVC sizes.

### Effect of Microplastics on M13 Phage

After 1 h, M13 phage activity decreased by 15–45% in TM buffer, while BM conditions showed a stronger reduction of 50–75% across all size fractions. At 24 h, inactivation increased to 40–50% in TM buffer and 55–80% in BM. By 168 h, 85–95% of phages were inactive in TM, while in BM, viability dropped by more than 95%, approaching complete inactivation.

In the presence of PET, M13 phage activity was reduced after 1 h, with a 25–30% decrease in TM buffer and a 45–75% reduction in BM across all size fractions. At 24 h, phage activity further declined, showing a 40–60% decrease in TM buffer and 70–75% in BM. By 168 h, the effect was consistent across all PET sizes, resulting in 85–95% inactivation in TM buffer and 95–100% in BM.

M13 phage activity decreased by 10–30% in both TM buffer and BM across all PS size fractions after 1 h. At 24 h, phage activity declined further, showing a 30–45% reduction in TM buffer, while BM showed a stronger reduction (65–80%) across all sizes except PS4 (<100 μm), which remained around 40%. By 168 h, phage activity declined to 85–95% in the TM buffer, while BM showed more than 95% inactivation with only little variation across sizes.

After 1 h, M13 phage activity decreased by 20–35% in TM buffer and 10–30% in BM across all sizes. At 24 h, inactivation increased to 35–55% in TM buffer and 55–70% in BM across all size fractions. By 168 h, 90–95% of phages were inactivated in TM, while in BM, viability dropped by more than 95% to almost complete inactivation across all PVC sizes.

### Why Do Microplastics Affect Virions?

We have already shown that the virions strongly adsorb to plastic surfaces, including PS, as evidenced by phage loss in PS containers.^33^ Virions blocking the hydrophobic surfaces resulted in a more thermodynamically preferred state of the system. We have also shown that the heteroaggregation of virions and microplastic particles is governed by electrostatic interactions.^24^ Leachables present in plastics (e.g., plasticizers) have a smaller impact on the studied viruses.^24^ Here, we aim to understand the mechanism better. A more comprehensive quantitative analysis of the impact of experimental conditions and the relationships between key factors is examined in the following section.

### Heteroaggregation in the Multivariate Approach

Linear regression was used to analyze the impact of variables in the experiment. This approach allows the description of the dependent variable *y* with the independent variables *X*. The β parameters are found with the ordinary least-squares method. ε is a random variable.

y=Xβ+ε

$$y=\left[\begin{array}{l} y_{1}\\ \vdots \\ y_{i} \end{array}\right],\;X=\left[\begin{array}{lll} x_{1,1} & \cdot \cdot \cdot & x_{1j}\\ \vdots & \ddots & \vdots \\ x_{i1} & \cdot \cdot \cdot & x_{ij} \end{array}\right],\;\beta =\left[\begin{array}{l} \beta_{1}\\ \vdots \\ \beta_{j} \end{array}\right],\;\varepsilon =\left[\begin{array}{l} \varepsilon_{1}\\ \vdots \\ \varepsilon_{i} \end{array}\right]$$

The model can be further modified by introducing functions of the dependent variable and the independent variables (including nonlinear functions). Hence, we look for an estimation of the observed phenomena with the following equation

$$f_{y}\left(y_{pfu}\;\right)=\sum {\beta_{i}f}_{i}\left({var}_{i}\right)$$

*y*_pfu_—concentration of bacteriophages in phage-forming units; var_*i*_—independent variable selected for the regression.

Linear regression guided by functions based on physicochemical theories of aggregation could show significant parameters of aggregation. However, linear regressions conducted with physicochemical variables (e.g., zeta potential of phages, zeta potential of polymers, and radius of plastic particles) were not satisfactory. The challenges arose due to the high correlation of the significant variables, the non-normal distribution of the residuals, and not passing the diagnostic tests. For these reasons, conclusions from the models were pointless even if *R* ^2^ reached over 80%. Instead, we present a regression based on the experiment design.

Table 2 presents a joint regression of variables: type of phage, size sample of a polymer, type of polymer, and presence of biomedium. The dependent variable was defined as the percentage of titer (PFU/mL) of a control experiment (*y*_percentage_). More details of the regression, such as basic diagnostics, residuals’ distribution, and analogous regressions for log–linear structure (natural logarithm of *y*_percentage_ as a dependent variable), can be found in Supporting Information (Figures S14–S19).

**Table 2:** Linear Regression Results for *y*_percentage_ as a Dependent Variable^a^

	1 h (*R* ^2^ = 49.2%)			24 h (*R* ^2^ = 64.4%)			168 h (*R* ^2^ = 88.6%)		
variable	β	std. err.	*p*-value	Β	std. err.	*p*-value	β	std. err.	*p*-value
constant	114.1	6.0	0.00	103.4	4.9	0.00	86.9	3.2	0.00
BM	–3.3	2.7	0.21	–10.7	2.2	0.00	–2.5	1.4	0.08
M13	–19.7	3.3	0.00	–8.4	2.7	0.00	–2.9	1.7	0.10
T4	–17.7	3.3	0.00	–6.3	2.7	0.02	3.0	1.7	0.08
PS	9.8	3.9	0.01	–9.2	3.2	0.01	–4.6	2.1	0.03
PVC	9.9	3.9	0.01	–13.7	3.2	0.00	–4.9	2.1	0.02
PE	–3.3	3.9	0.41	–13.1	3.2	0.00	–5.0	2.1	0.02
size 1	–30.7	6.6	0.00	–39.7	5.5	0.00	–76.5	3.5	0.00
size 2	–28.6	6.6	0.00	–39.5	5.5	0.00	–74.1	3.5	0.00
size 3	–30.9	6.6	0.00	–41.1	5.5	0.00	–74.0	3.5	0.00
Size 4	–34.3	6.6	0.00	–34.8	5.5	0.00	–74.3	3.5	0.00

a Due to colinearity, results for MS2 and PET in TM are taken as a baseline.

Since regressions with physicochemical variables did not provide reliable results, we tried an alternative approach.^24^ The interactions between the type of phage and the zeta potential of the polymer were used as explanatory variables. Here, interactions with the biomedium were added, as well. The zeta potential of polymers was used as a second-order polynomial (β_1_zeta_polymer + β_2_zeta_polymer^2^). Regressions with one of these polynomial terms were not successful. To clarify, by the interaction between, for example, biomedium, T4, and zeta-potential of polymer, we understand a multiplication of binary variables describing whether biomedium was used, binary variable whether T4 was used, and zeta-potential of the polymer. The results are presented in the Supporting Information (Figures S20–S30). The insignificant variables were removed from the regressions. Those results were similar to or minimally better in terms of *R* ^2^. Such model construction is doubtful as the variables were categorized as the type of phage and the presence of biomedium, leaving only the zeta potential of the polymer to be described by a numerical value. Only the log–linear model for 24 h passed the diagnostic tests (α = 0.05). Nonetheless, variance inflation factors and correlation between variables were very high (|correlations| > 0.2). Concluding on a physical meaning of the estimated β parameters is not appropriate.

## Discussion

This study demonstrates that exposure to microplastics significantly affects bacteriophage viability with notable differences based on phage type, microplastic composition, and environmental conditions. Across all tested conditions, phage inactivation was more pronounced in biomedium (BM) than in TM buffer, suggesting that organic matter from bacterial debris is critical in influencing the phage suspension stability. Additionally, while no clear correlation between microplastic size and phage inactivation was consistently observed, distinct trends emerged based on the plastic type and incubation time.

### Microplastic Influence on Phage Viability

MS2, T4, and M13 phages exhibited reduced viability when exposed to microplastics, particularly over extended incubation periods. The most substantial inactivation occurred at 168 h, where 85–100% reductions were observed across different microplastic types and sizes. These findings align with our previous reports indicating that viral particles can adsorb onto microplastic surfaces, leading to reduced bioavailability and possible degradation.^24^ The more substantial reduction in BM suggests phage aggregation with organic matter, further enhancing attachment to microplastics and leading to physical removal or reduced infectivity. The role of microplastic composition was evident, with PVC and PET causing the greatest phage inactivation, while PE and PS had comparatively lesser effects. This difference may stem from variations in surface properties, such as hydrophobicity, surface charge, and leachable chemical additives.

### Phage-Specific Sensitivity to Microplastics

Phage susceptibility varied among the tested viruses. MS2, a small icosahedral phage, exhibited rapid inactivation, particularly in BM, suggesting that its surface charge and compact structure make it more susceptible to adsorption and deactivation. In contrast, the T4 phage, a tailed bacteriophage, exhibited greater stability of the colloidal suspension, possibly due to its larger size and more complex capsid structure, which may increase the resilience to adsorption or chemical interactions. M13 phage, a filamentous phage, showed intermediate susceptibility, with higher inactivation rates in BM, which could be attributed to its elongated morphology, increasing surface interaction with microplastics.

The observed phage inactivation likely results from a combination of direct adsorption onto microplastic surfaces, chemical interactions with plastic additives, and indirect effects from organic matter in BM. The greater inactivation in BM suggests that organic components enhance aggregation, altering the phage mobility and availability.

### Heteroaggregation in the Multivariate Approach

The results of all regressions show that the dependence of the activity of phages on the size of the microplastic used in the experiment is unclear. Differences between the parameters of the coarse models (Table 2) were within the standard errors of estimation of the parameters’ values. According to the BET analysis (Table S1), the segregation of microplastic particles with sieves is divided into microplastics according to size in most cases. However, the calculated size of the particles was different for different polymers. Linear regressions that considered sizes calculated according to the BET method did not provide convincing results, showing only sets of highly correlated variables with negative diagnostic tests.

The results of the calculations do not deny our previous conclusions.^24^ Some differences occurred (e.g., the impact of PE on the deactivation of phages is much more significant in this study than in the previous one), but the ζ-potential is still the most probable factor of aggregation. A better approximation of the model using categorical variables describing bacteriophages instead of their zeta potential suggests that the nonuniform distribution of the charge (the phenomenon present in the case of phages) may be crucial in predicting heteroaggregation. This may also be a factor that did not allow the regression of models that attempted to incorporate DLVO theory^34^ and first- or second-order kinetics.

We also hypothesize that the process in the early stages might be kinetically controlled by the efficiency and frequency of the collisions. The frequency of collisions might be estimated based on Smoluchowski’s shear-induced collision rate;^35^ however, the efficiency of the collisions was unknown. After 1 and 24 h, the differences between the effects of microplastics in TM and BM were large. This was most probably caused by the interactions of phages or phages and plastic particles, with bacterial debris in the BM. Similar behavior was observed in hollow, spherical glass beads dispersed in an aqueous glycerin solution when high molecular weight poly­(ethylene oxide) was used as the flocculating agent. The collision efficiency increased with the increase in the thickness of the polymer layer caused by increasing polymer concentration and molecular weight.^36^ However, after 7 days of incubation with microplastics, the differences between phage titers in TM and BM were less pronounced, suggesting that at this stage, the process was thermodynamically controlled. It was likely that multivariate analysis could not capture such a change.

### Environmental Implications

This study highlights the significant environmental consequences of microplastic contamination, particularly its impact on bacteriophage dynamics in marine ecosystems. As key regulators of microbial populations, bacteriophages influence biogeochemical cycles and ecosystem stability. Their adsorption and inactivation on microplastic surfaces, especially in organic-rich environments, suggest that microplastics may act as viral sinks, disrupting phage-mediated bacterial control. Such interference could drive shifts in microbial diversity and pathogenicity, altering community structures in ways that are difficult to predict. Additionally, microplastics may serve as vectors for phage transport, enabling viruses to hitchhike to new bacterial environments, where they could disrupt existing microbial homeostasis and introduce unforeseen ecological imbalances. Understanding these interactions is crucial for assessing the broader consequences of microplastic pollution on microbial ecosystems.

## Conclusion

This study demonstrates that microplastics significantly reduce the availability of active bacteriophages with organic matter further amplifying this effect. Even at lower concentrations than those found in marine environments, organic debris in biomedium led to substantial decreases in phage titers in the presence of microplastics, suggesting that such interactions could be highly relevant in natural ecosystems.

Attempts to identify the primary drivers of phage aggregation and inactivation using linear regression were inconclusive due to the high correlation of influencing factors, limiting the ability to isolate specific relations. However, coarse analysis revealed that microplastic size was insignificant, while the zeta potential could have an important role, reinforcing previous findings. The observed deviation from classical DLVO theory possibly originated from an anisotropic charge distribution on bacteriophages, and specific interactions between, for instance, surface proteins, microplastics, and organic matter may govern the heteroaggregation behavior. A more advanced supervised machine learning approach could provide deeper insight into the interplay of these physicochemical factors, though it would require a broader data set and more diverse experimental conditions.

Beyond these physicochemical insights, our findings suggest that microplastic pollution may have far-reaching ecological consequences. By disrupting phage-bacteria homeostasis, microplastics may alter microbial diversity and biogeochemical cycling, potentially impacting nutrient fluxes and ecosystem stability. Additionally, microplastics could act as vectors for viral transport, facilitating phage migration to new bacterial populations, including vertical movement within aquatic environments. These processes may influence microbial evolution, alter host–pathogen interactions, and reshape ecosystem dynamics in unpredictable ways.

Future research should focus on mechanistic studies to dissect how plastic additives, surface properties, and environmental conditions affect the viral stability. Expanding investigations to different bacterial hosts and environmental matrices will be critical for assessing the broader ecological implications of microplastic-driven viral perturbations. Given the increasing prevalence of plastic pollution, understanding its impact on microbial networks is essential for predicting long-term disruptions in aquatic ecosystems.

## Supplementary Material







## Data Availability

All data related to the presented results are available from the RepOD repository under https://doi.org/10.18150/PT7DM5.

## Notes

BET measurement results, tabularized results of phage titration experiments (1 h, 24 h, 168 h), changes in phage titer plotted in time, description of multivariate analysis, detailed results of linear regressions, and Python scripts (PDF)
Database with the description of the variables (XLSX)
List of variables mentioned in the database (TXT)

## Abbreviations

PE: polyethylene
PET: polyethylene terephthalate
PS: polystyrene
PVC: polyvinyl chloride
PP: polypropylene
PU: polyurethane
PFU: plaque-forming units
BET: Brunauer, Emmet, Teller
BM: biomedium
DLVO: Derjaguin–Landau–Verwey–Overbeek.
