# Supplementary material for: Microplastics of Broad Size Range Reduce Bacteriophage Activity in Aqueous Environments
Source: J Phys Chem B. 2025 Jun 10;129(24):6100–10. doi: 10.1021/acs.jpcb.5c01316 (PMC12183763; doi:10.1021/acs.jpcb.5c01316)
Supplement: Supplementary file 1 [file jp5c01316_si_001.pdf]

Supporting Information

Microplastics of Broad Size Range Reduce Bacteriophage Activity in Aqueous Environments

Enkhlin Ochirbat [1‡], Rafał Zbonikowski [1‡], Michał Folga [1,2], Magdalena Bonarowska [1], Jan Paczesny[1,]*

eochirbat@ichf.edu.pl, rzbonikowski@ichf.edu.pl, michal.folga99@gmail.com, mbonarowska@ichf.edu.pl, jpaczesny@ichf.edu.pl

[1] Institute of Physical Chemistry, Polish Academy of Sciences, Kasprzaka 44/52, 01-224 Warsaw, Poland

[2] Warsaw University of Technology, Faculty of Chemistry, Noakowskiego 3, 00-664, Warsaw, Poland

[‡] These authors contributed equally

*** Corresponding Author:** Jan Paczesny jpaczesny@ichf.edu.pl, +48 22 343 2071

1. Extra experimental results

1.1. Microplastic BET characterization

Surface areas, total pore volumes, and average pore size of polymers were determined by Micromeritics ASAP2020. The weight of the samples was ~0.3 g. Before measurements, the samples were degassed in a vacuum at 343 K for 5 hours to clean their surface. The adsorption process was carried out at a temperature of liquid nitrogen (77 K), and krypton was used as an adsorbent instead of the commonly used nitrogen. For extremely low surface area samples (which is what the polymers discussed in this paper are), the number of non-adsorbed gas molecules at adsorption equilibrium can exceed the number of molecules adsorbed on the sample, which will hamper the sample's accurate measurement of gas uptake. Because of this effect, the typical surface area detection limit for N_2 physisorption at 77 K is assumed to be about 1 m^2. This detection limit may be significantly reduced by using krypton adsorption analysis at the same temperature that krypton is below its triple point and where its saturation

pressure is 2.32 mbar, i.e., ~430 times lower than p_{sat} of N_2. It follows that at any given relative gas pressure, the absolute pressure of Kr is 430 times lower than that of N_2. This also means that the density of Kr in the free space is proportionally lower, which significantly improves the detection limit for Kr. The surface area calculation was carried out following the theory of Brunauer, Emmet, and Teller (BET) as recommended by the IUPAC for low-surface substances [1]. The BET model was applied in the range of p/p_0 from 0.05 to 0.30, and the resulting BET plots showed linearity (the measurement error level was 5%).

Table S1. Results of BET analysis.

Polymer	size (*variable's name*)	*bet_area* [m^2 g^{-1}]	*tplot_area* [m^2 g^{-1}]	*pore_volume* [m^3 g^{-1}]	*pore_width* [Å]	*bet_size* [µm]
PS	Size 1 (*size_a*)	0.07480	0.07900	0.000016	19.70	80.8
	Size 2 (*size_b*)	0.07170	0.06360	0.000019	23.25	108.2
	Size 3 (*size_c*)	0.12105	0.13130	0.000036	22.00	60.4
	Size 4 (*size_d*)	0.27355	0.25100	0.000071	21.55	26.1
PVC	Size 1 (*size_a*)	0.10770	0.12320	0.000024	19.70	66.5
	Size 2 (*size_b*)	0.07830	0.08120	0.000021	23.10	77.5
	Size 3 (*size_c*)	0.14640	0.18690	0.000041	20.65	38.0
	Size 4 (*size_d*)	0.21970	0.22120	0.000064	23.65	28.2
PET	Size 1 (*size_a*)	0.06600	0.06480	0.000020	22.40	140.2
	Size 2 (*size_b*)	0.06310	0.05860	0.000018	23.20	125.5
	Size 3 (*size_c*)	0.09930	0.08820	0.000031	21.45	69.6
	Size 4 (*size_d*)	0.13360	0.13200	0.000040	21.45	55.2
PE	Size 1 (*size_a*)	0.04655	0.04495	0.000013	23.55	348.1
	Size 2 (*size_b*)	0.05930	0.06560	0.000015	27.20	127.3
	Size 3 (*size_c*)	0.12210	0.11360	0.000029	23.10	54.7
	Size 4 (*size_d*)	0.16500	0.14790	0.000039	22.70	49.7

Table S2. Changes in phage titer following incubation with microplastics (PE, PET, PS, PVC) of different size fractions ("1": 1 – 5 mm, "2": 500 – 1000 µm, "3": 100 – 500 µm, "4": < 100 µm) for 1 hour in TM buffer and biomedium are presented. The number of active phages is expressed as a percentage of the initial titer.

		TM buffer			*Biomedium*		
Polymer	Size	T4	M13	MS2	T4	M13	MS2
PE	1	66.67 ± 3.60 **	67.16 ± 3.50 **	85.08 ± 2.63	60.53 ± 4.77 **	29.11 ± 1.99 ***	89.32 ± 2.93
	2	75.27 ± 3.68	53.73 ± 2.95 ***	83.98 ± 2.44 **	61.84 ± 4.59 **	44.30 ± 2.06 ***	93.20 ± 3.80
	3	64.52 ± 3.00 **	85.07 ± 5.73 **	81.77 ± 2.15 ***	55.26 ± 3.42 **	31.65 ± 3.07 ***	87.38 ± 3.80 *
	4	52.69 ± 3.40 ***	67.16 ± 3.50 **	40.33 ± 3.07 ***	75.00 ± 4.09 *	25.32 ± 2.13 ***	98.06 ± 3.32
PET	1	73.12 ± 3.24 *	73.13 ± 6.03 *	88.40 ± 2.01 *	59.21 ± 4.12 **	39.24 ± 2.93 ***	96.12 ± 3.84
	2	78.49 ± 4.32 **	76.12 ± 3.70 **	85.08 ± 1.70 *	63.16 ± 3.87 **	53.16 ± 2.72 ***	104.85 ± 3.33
	3	76.34 ± 4.04 **	74.63 ± 3.61 **	87.29 ± 2.54 **	55.26 ± 3.62 ***	24.05 ± 2.10 ***	73.79 ± 2.66 ***
	4	67.74 ± 3.31 **	71.64 ± 4.09 *	61.88 ± 1.88 **	60.53 ± 3.55 ***	24.05 ± 2.10 ***	85.44 ± 3.48 *
PS	1	54.84 ± 3.37 ***	71.64 ± 3.61 *	91.71 ± 2.25 *	69.74 ± 4.84 **	82.28 ± 3.42	92.23 ± 4.13
	2	56.99 ± 3.27 **	73.13 ± 3.50 *	91.71 ± 2.01 *	73.68 ± 3.79 **	78.48 ± 3.60 *	81.55 ± 3.31 *
	3	62.37 ± 3.07 ***	73.13 ± 3.22 ***	91.71 ± 2.46	68.42 ± 3.63 ***	86.08 ± 3.61	96.12 ± 4.04
	4	63.44 ± 2.96 **	80.60 ± 3.22 ***	87.29 ± 3.68 ***	72.37 ± 4.19 ***	88.61 ± 3.61 *	99.03 ± 3.96
PVC	1	68.82 ± 4.17 *	77.61 ± 3.49 *	87.29 ± 1.55	56.58 ± 4.06 ***	92.41 ± 3.25	88.35 ± 2.90 **
	2	75.27 ± 3.81 **	65.67 ± 3.74 **	93.92 ± 2.47	68.42 ± 4.01 ***	89.87 ± 3.76	90.29 ± 3.43
	3	77.42 ± 4.68 **	76.12 ± 3.70 **	91.71 ± 2.46 *	76.32 ± 4.01 *	78.48 ± 3.95 *	81.55 ± 3.31 **
	4	95.70 ± 5.03 *	67.16 ± 2.92 ***	74.59 ± 3.39 **	69.74 ± 4.20 *	68.35 ± 3.42 **	77.67 ± 2.58 ***

Table S3. Changes in phage titer following incubation with microplastics (PE, PET, PS, PVC) of different size fractions ("1": 1 – 5 mm, "2": 500 – 1000 µm, "3": 100 – 500 µm, "4": < 100 µm) for 24 hours in TM buffer and biomedium are presented. The number of active phages is expressed as a percentage of the initial titer.

		TM buffer			*Biomedium*		
Polymer	Size	T4	M13	MS2	T4	M13	MS2
PE	1	46.24 ± 2.72<br>***	50.75 ± 4.14<br>***	37.02 ± 1.30<br>***	32.89 ± 2.85<br>***	40.51 ± 2.02<br>***	40.78 ± 1.53<br>***
	2	47.31 ± 2.38<br>***	47.76 ± 4.59<br>***	33.70 ± 1.38<br>***	30.26 ± 1.88<br>***	20.25 ± 1.74<br>***	37.86 ± 2.08<br>***
	3	48.39 ± 3.09<br>***	61.19 ± 3.77	35.36 ± 1.08<br>***	36.84 ± 2.81<br>***	20.25 ± 1.74<br>***	39.81 ± 1.90<br>***
	4	45.16 ± 2.84<br>***	53.73 ± 4.45<br>***	57.46 ± 2.34<br>***	53.95 ± 3.32<br>***	16.46 ± 1.68<br>***	57.28 ± 2.05<br>***
PET	1	43.01 ± 3.85<br>***	58.21 ± 3.18<br>***	38.67 ± 1.78<br>***	31.58 ± 2.71<br>***	21.52 ± 2.23<br>***	42.72 ± 1.85<br>***
	2	37.63 ± 2.73<br>***	47.76 ± 2.84<br>***	54.14 ± 1.35<br>***	30.26 ± 2.80<br>***	22.78 ± 1.67<br>***	54.37 ± 2.20<br>***
	3	31.18 ± 1.45<br>***	50.75 ± 2.46<br>***	35.36 ± 1.48<br>***	28.95 ± 3.10<br>***	24.05 ± 1.75<br>***	42.72 ± 1.85<br>***
	4	53.76 ± 3.60<br>***	40.30 ± 2.69<br>***	52.49 ± 2.06<br>***	32.89 ± 3.52<br>***	30.38 ± 1.86<br>***	69.90 ± 2.10<br>*
PS	1	49.46 ± 3.98<br>***	61.19 ± 4.23<br>**	37.57 ± 1.40<br>***	40.79 ± 3.23<br>***	18.99 ± 1.89<br>***	41.75 ± 1.82<br>***
	2	46.24 ± 2.34<br>***	58.21 ± 2.87<br>***	38.67 ± 1.71<br>***	35.53 ± 3.25<br>***	29.11 ± 2.58<br>***	46.60 ± 1.68<br>***
	3	38.71 ± 2.39<br>***	56.72 ± 2.94<br>***	53.04 ± 1.17<br>***	39.47 ± 3.28<br>***	18.99 ± 1.49<br>***	55.34 ± 1.92<br>***
	4	54.84 ± 3.07<br>**	68.66 ± 2.90<br>***	61.88 ± 1.41<br>***	31.58 ± 2.43<br>***	53.16 ± 2.18<br>***	63.11 ± 2.06<br>***
PVC	1	67.74 ± 3.31<br>**	67.16 ± 3.76<br>**	46.41 ± 2.20<br>**	56.58 ± 3.05<br>***	37.97 ± 1.89<br>***	56.31 ± 2.00<br>***
	2	65.59 ± 3.02<br>**	52.24 ± 3.48<br>***	82.87 ± 1.35<br>*	44.74 ± 2.93<br>***	37.97 ± 2.50<br>***	69.90 ± 2.10<br>**
	3	51.61 ± 4.21<br>***	62.29 ± 3.62<br>***	79.56 ± 1.96<br>***	42.11 ± 2.93<br>***	26.58 ± 1.78<br>***	68.93 ± 2.80<br>**
	4	60.22 ± 4.00<br>***	44.78 ± 2.33<br>***	38.67 ± 1.63<br>***	51.32 ± 3.02<br>***	34.18 ± 2.50<br>***	59.22 ± 2.08<br>***

Table S4. Changes in phage titer following incubation with microplastics (PE, PET, PS, PVC) of different size fractions ("1": 1 – 5 mm, "2": 500 – 1000 µm, "3": 100 – 500 µm, "4": < 100 µm) for 168 hours in TM buffer and biomedium are presented. The number of active phages is expressed as a percentage of the initial titer.

		TM buffer			*Biomedium*		
Polymer	Size	T4	M13	MS2	T4	M13	MS2
PE	1	9.35 ± 0.40 ***	3.73 ± 0.37 ***	0.61 ± 0.09 ***	0.38 ± 0.24 ***	1.65 ± 0.12 ***	7.28 ± 0.28 ***
	2	17.10 ± 0.91 ***	4.03 ± 0.36 ***	0.55 ± 0.07 ***	0.87 ± 0.31 ***	1.01 ± 0.17 ***	6.50 ± 0.28 ***
	3	8.28 ± 0.41 ***	12.84 ± 0.92 ***	1.27 ± 0.09 ***	12.76 ± 0.64 ***	1.52 ± 0.12 ***	2.82 ± 0.21 ***
	4	4.73 ± 0.26 ***	3.58 ± 0.35 ***	23.20 ± 1.01 ***	13.68 ± 0.68 ***	1.52 ± 0.24 ***	3.40 ± 0.23 ***
PET	1	8.06 ± 0.34 ***	14.03 ± 0.69 ***	0.50 ± 0.06 ***	12.89 ± 0.69 ***	1.52 ± 0.12 ***	1.46 ± 0.11 ***
	2	8.06 ± 0.36 ***	4.78 ± 0.28 ***	1.55 ± 0.11 ***	12.50 ± 0.60 ***	1.39 ± 0.12 ***	2.52 ± 0.18 ***
	3	6.34 ± 0.28 ***	4.48 ± 0.30 ***	1.38 ± 0.12 ***	12.63 ± 0.67 ***	1.65 ± 0.17 ***	2.52 ± 0.20 ***
	4	5.70 ± 0.37 ***	4.63 ± 0.30 ***	18.78 ± 1.00 ***	12.76 ± 0.67 ***	1.52 ± 0.20 ***	2.82 ± 0.19 ***
PS	1	3.76 ± 0.19 ***	4.78 ± 0.31 ***	0.39 ± 0.06 ***	10.79 ± 0.58 ***	1.39 ± 0.12 ***	0.42 ± 0.02 ***
	2	6.56 ± 0.39 ***	15.37 ± 0.60 ***	0.55 ± 0.07 ***	9.74 ± 0.54 ***	1.27 ± 0.16 ***	0.55 ± 0.02 ***
	3	8.60 ± 0.48 ***	4.33 ± 0.36 ***	2.98 ± 0.09 ***	13.68 ± 0.72 ***	2.15 ± 0.19 ***	17.48 ± 1.53 ***
	4	3.98 ± 0.24 ***	13.58 ± 0.56 ***	1.22 ± 0.08 ***	12.50 ± 0.64 ***	2.15 ± 0.15 ***	16.50 ± 1.69 ***
PVC	1	19.14 ± 0.72 ***	5.37 ± 0.26 ***	1.05 ± 0.11 ***	15.66 ± 0.64 ***	0.89 ± 0.14 ***	6.80 ± 0.24 ***
	2	32.26 ± 2.21 ***	4.48 ± 0.27 ***	39.78 ± 1.10 ***	12.76 ± 0.65 ***	1.14 ± 0.18 ***	5.92 ± 0.21 ***
	3	10.54 ± 0.51 ***	4.63 ± 0.30 ***	41.44 ± 1.31 ***	12.24 ± 0.58 ***	2.15 ± 0.22 ***	5.73 ± 0.22 ***
	4	5.16 ± 0.30 ***	7.16 ± 0.41 ***	0.66 ± 0.09 ***	12.76 ± 0.65 ***	2.15 ± 0.22 ***	12.62 ± 1.27 ***

M13

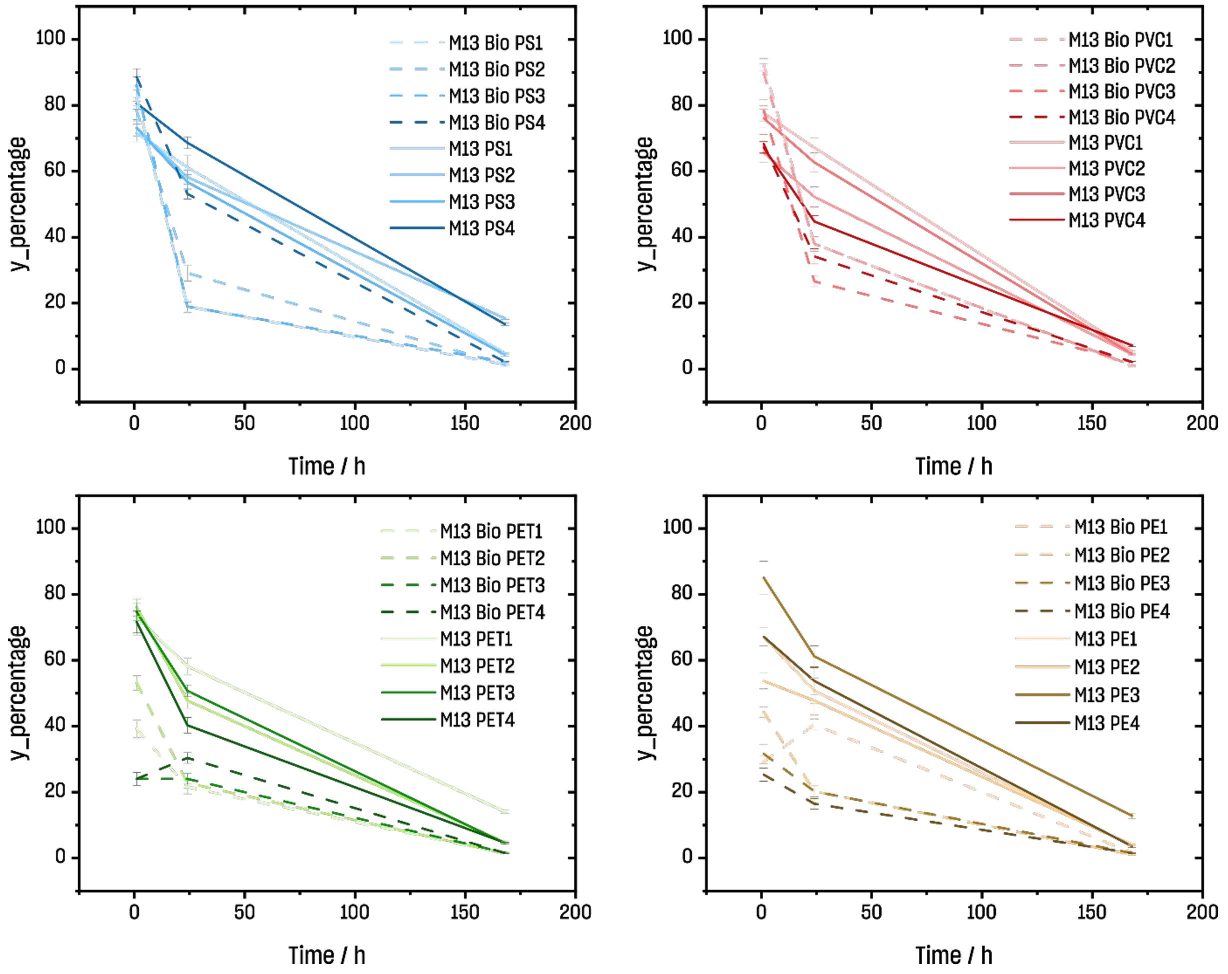

Figure S1. Experimental data for M13. Time dependence of active bacteriophages in TM buffer or biomedium (BM) for various polymer sizes (POLYMER1-4), as a percentage of active bacteriophages in control experiments for 1 h.

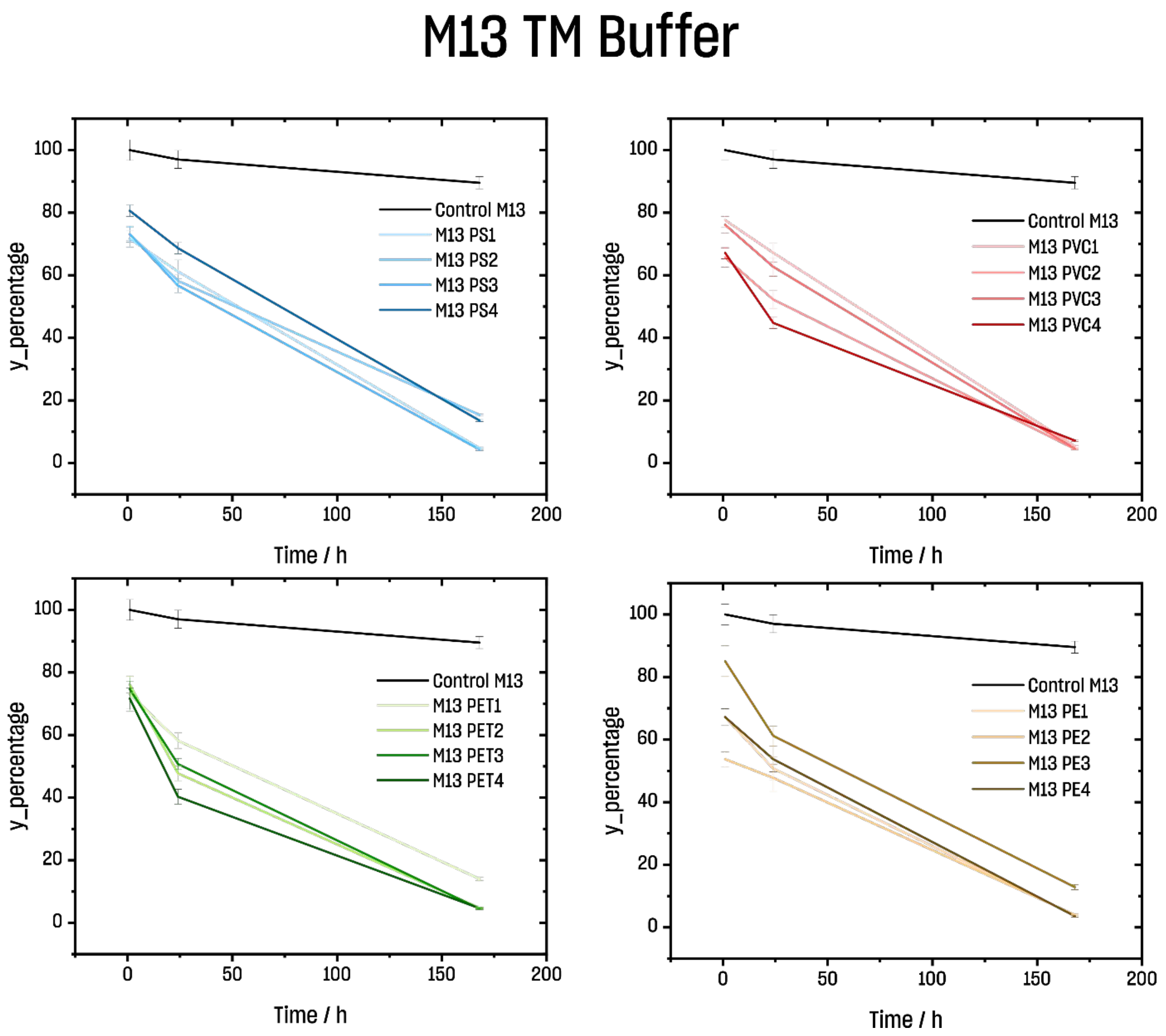

Figure S2. Experimental data for M13. Time dependence of active bacteriophages in TM buffer for various polymer sizes (POLYMER1-4), as a percentage of active bacteriophages in control experiments for 1 h.

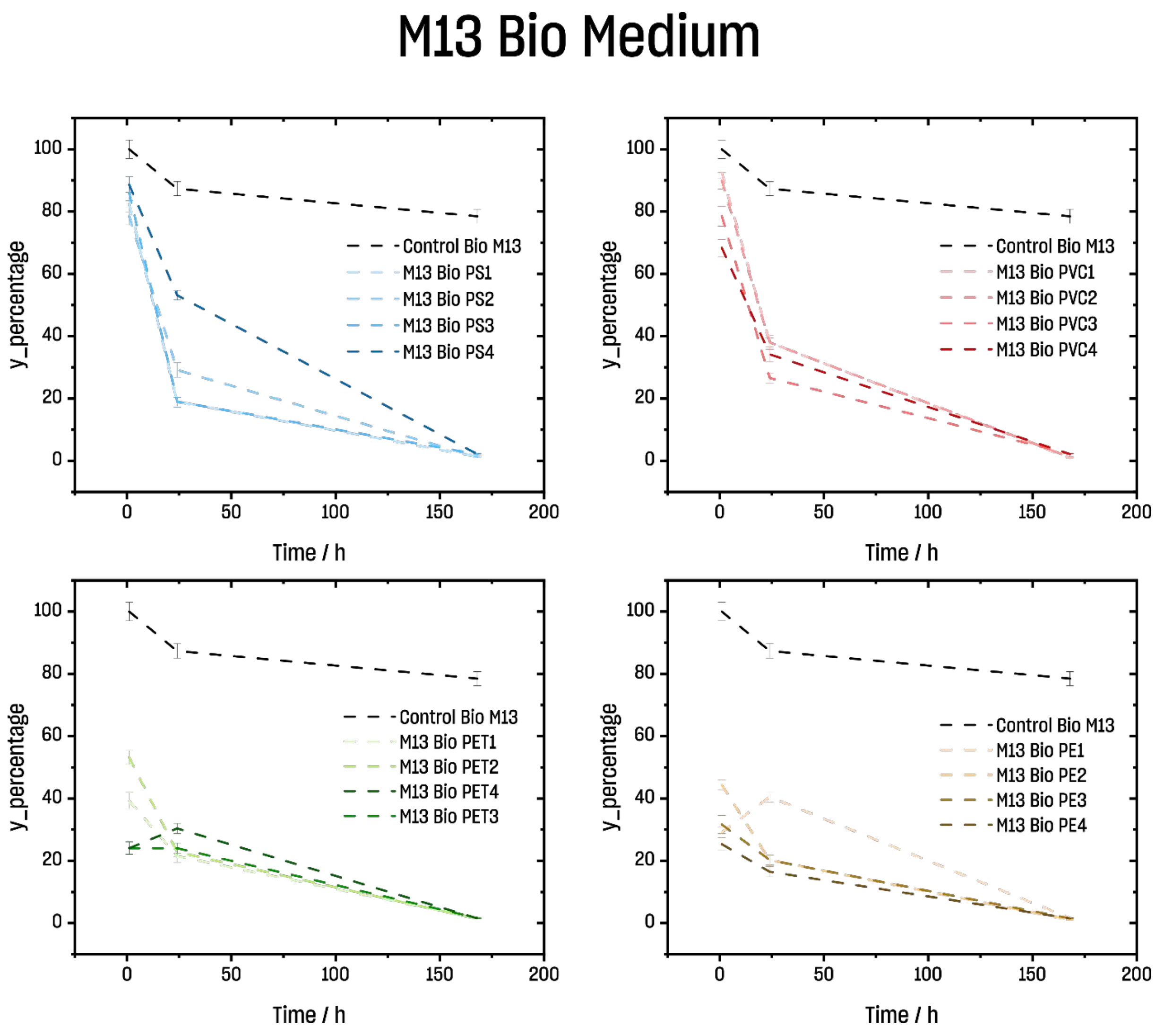

Figure S3. Experimental data for M13. Time dependence of active bacteriophages in biomedium (BM) for various polymer sizes (POLYMER1-4), as a percentage of active bacteriophages in control experiments for 1 h.

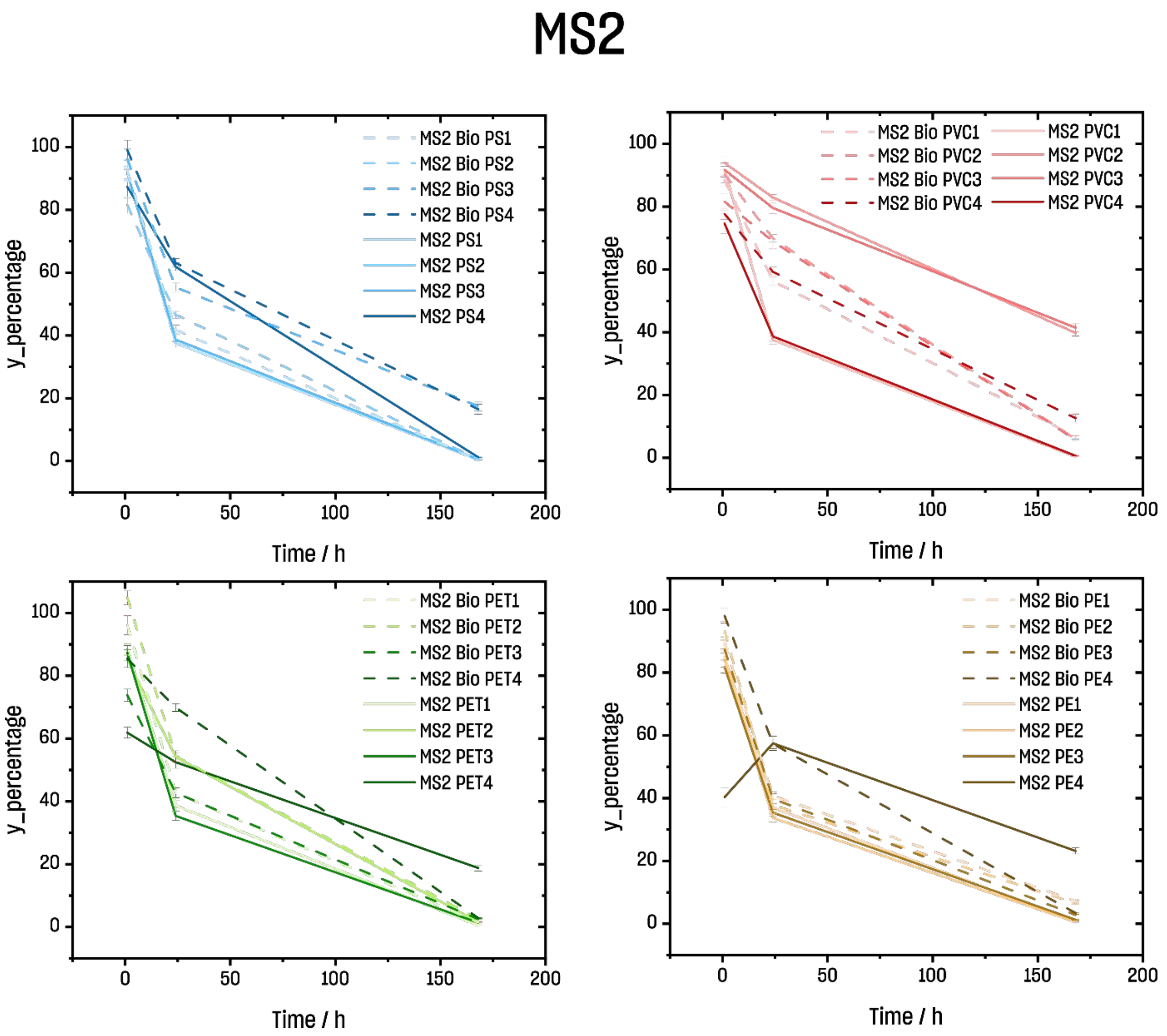

Figure S4. Experimental data for MS2. Time dependence of active bacteriophages in TM buffer or biomedium (BM) for various polymer sizes (POLYMER1-4), as a percentage of active bacteriophages in control experiments for 1 h.

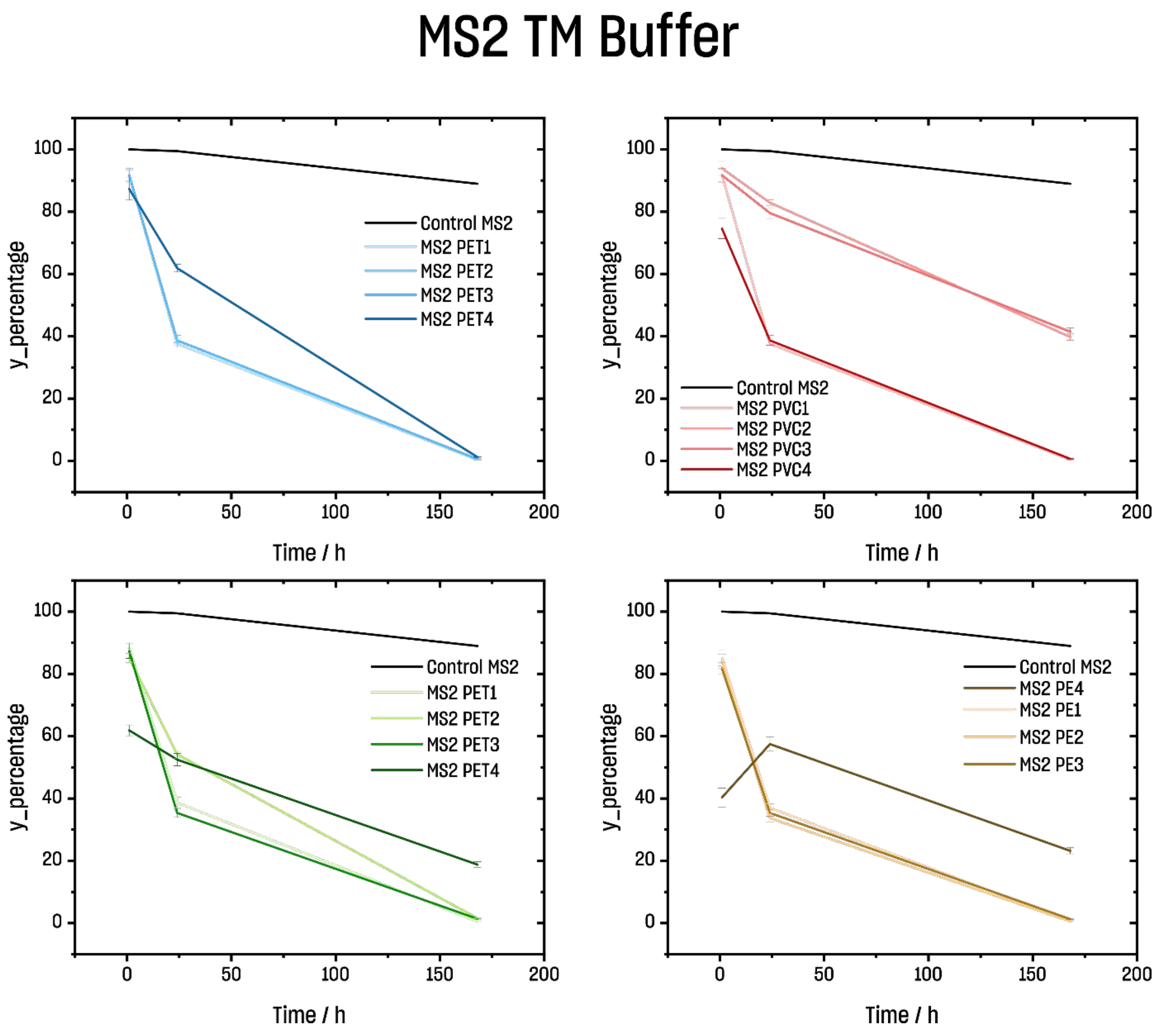

Figure S5. Experimental data for MS2. Time dependence of active bacteriophages in TM buffer for various polymer sizes (POLYMER1-4), as a percentage of active bacteriophages in control experiments for 1 h.

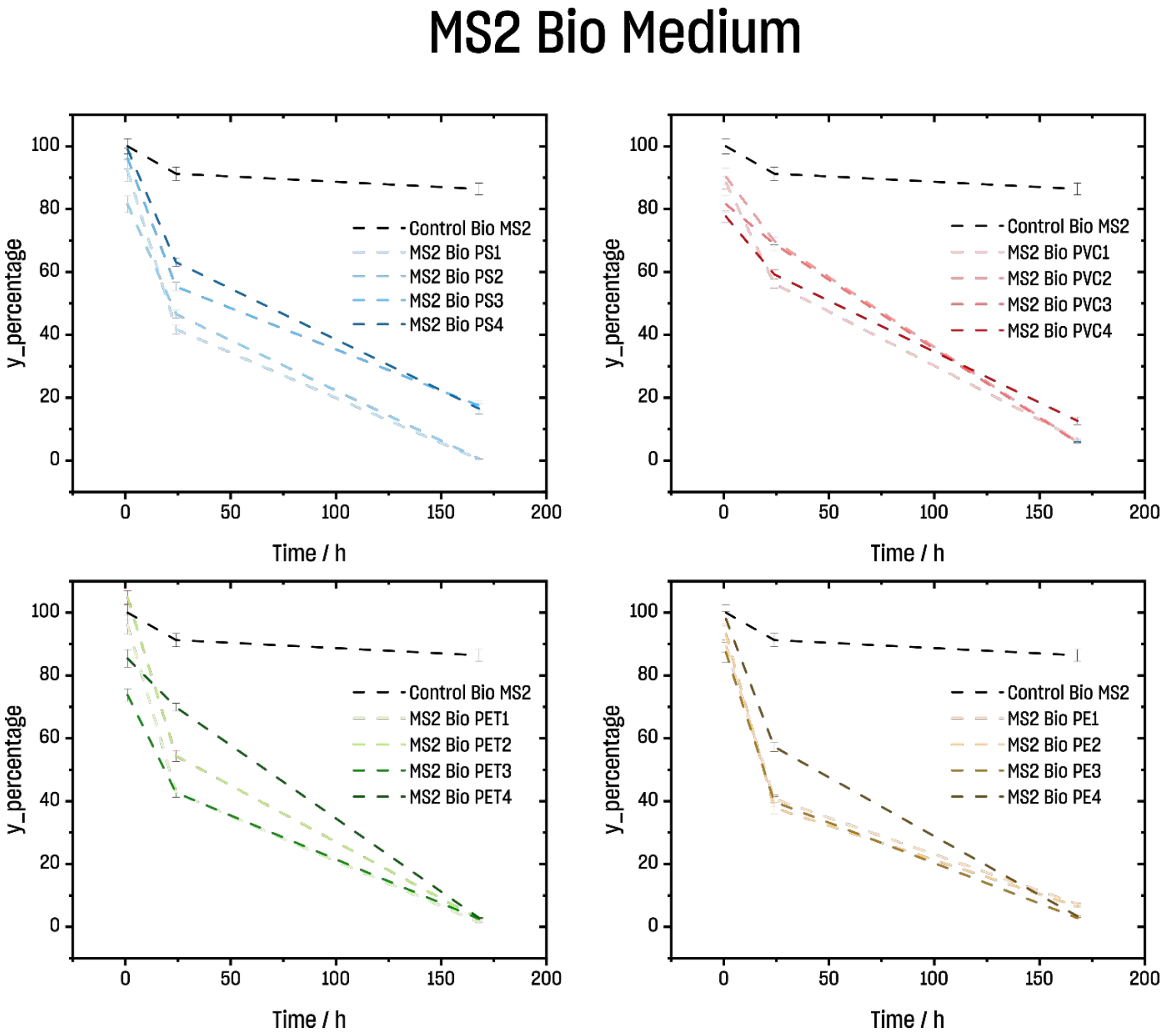

Figure S6. Experimental data for MS2. Time dependence of active bacteriophages in biomedium (BM) for various polymer sizes (POLYMER1-4), as a percentage of active bacteriophages in control experiments for 1 h.

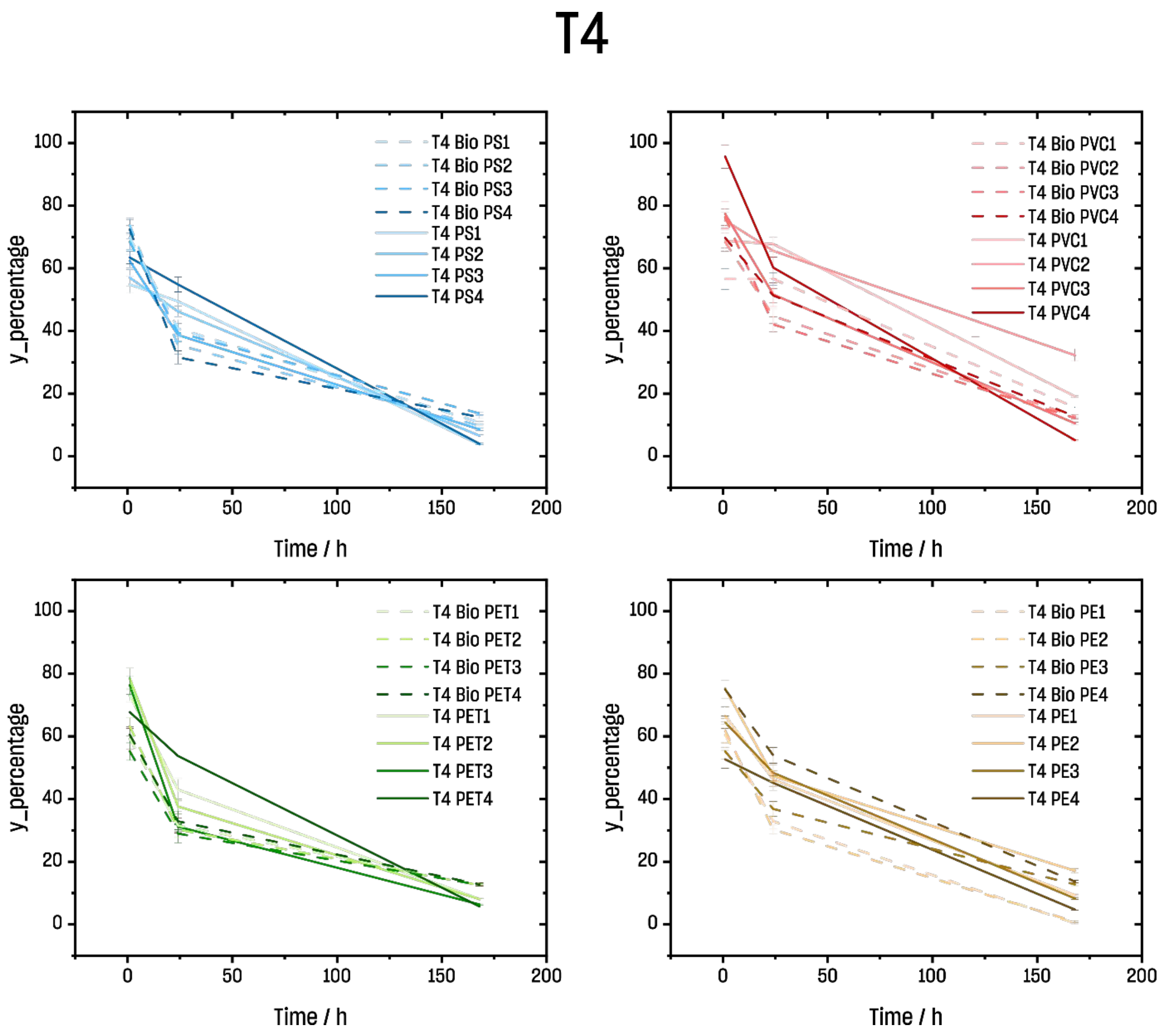

Figure S7. Experimental data for T4. Time dependence of active bacteriophages in TM buffer or biomedium (BM) for various polymer sizes (POLYMER1-4), as a percentage of active bacteriophages in control experiments for 1 h.

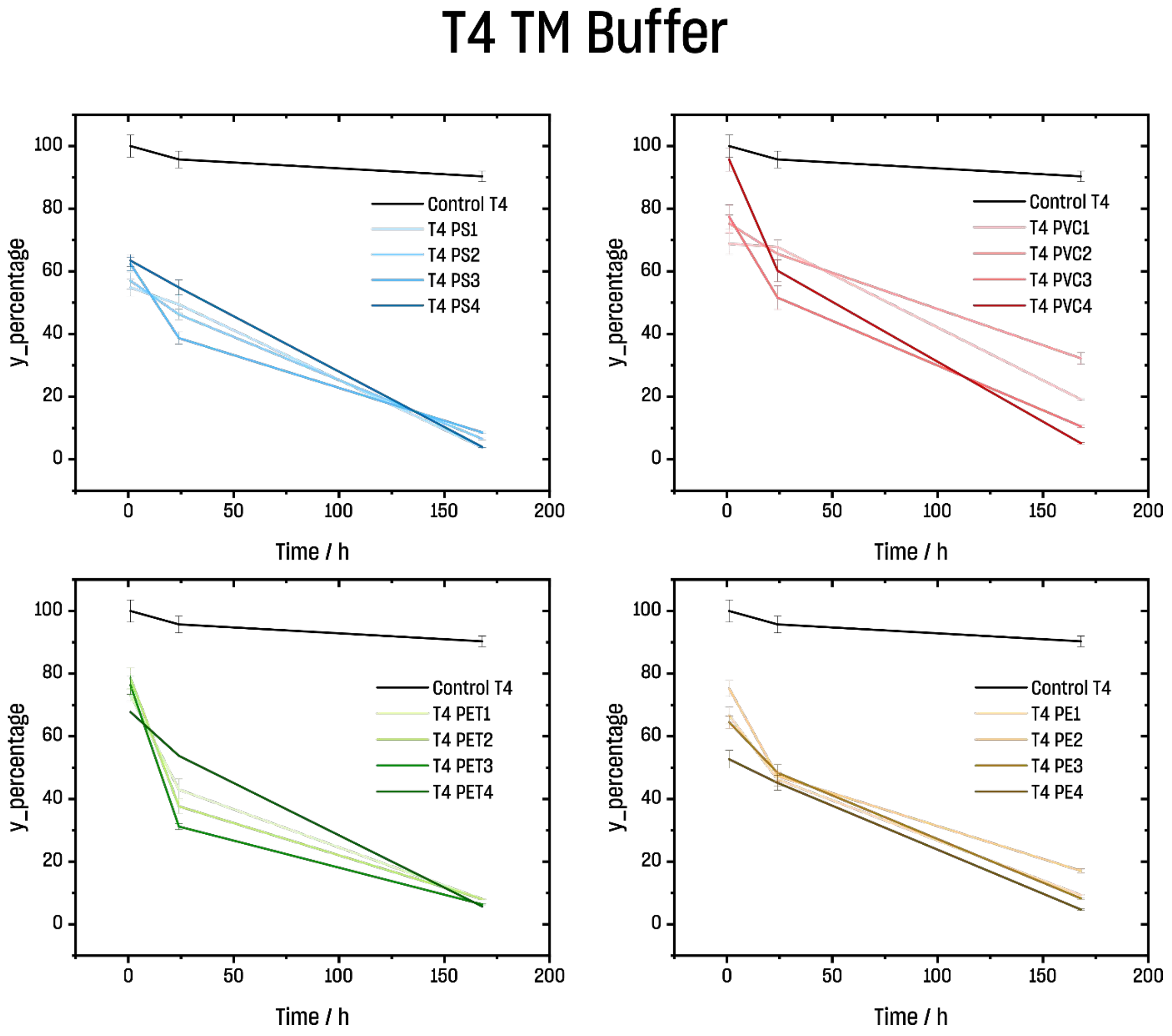

Figure S8. Experimental data for T4. Time dependence of active bacteriophages in TM buffer for various polymer sizes (POLYMER1-4), as a percentage of active bacteriophages in control experiments for 1 h.

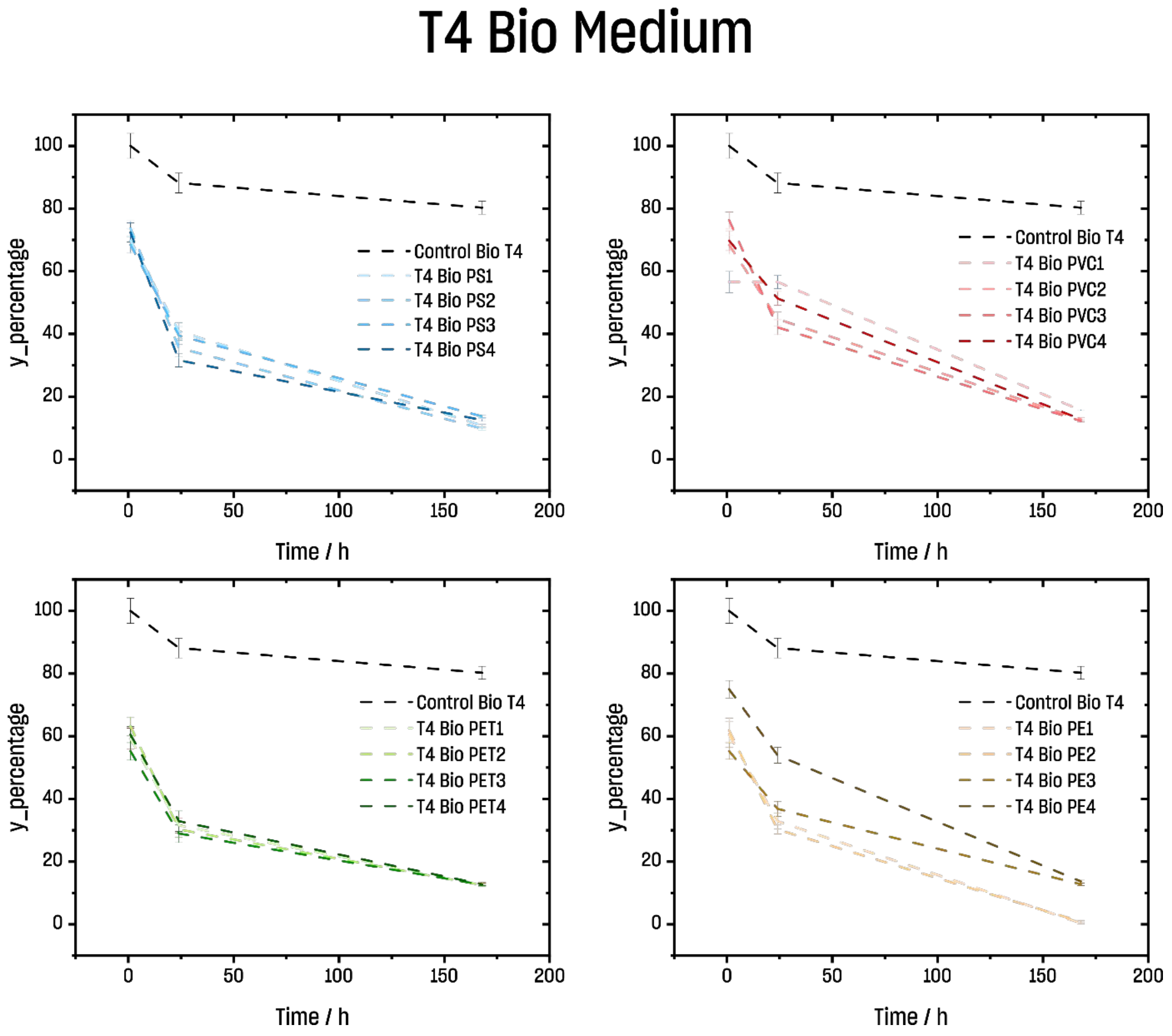

Figure S9. Experimental data for T4. Time dependence of active bacteriophages in biomedium (BM) for various polymer sizes (POLYMER1-4), as a percentage of active bacteriophages in control experiments for 1 h.

PS

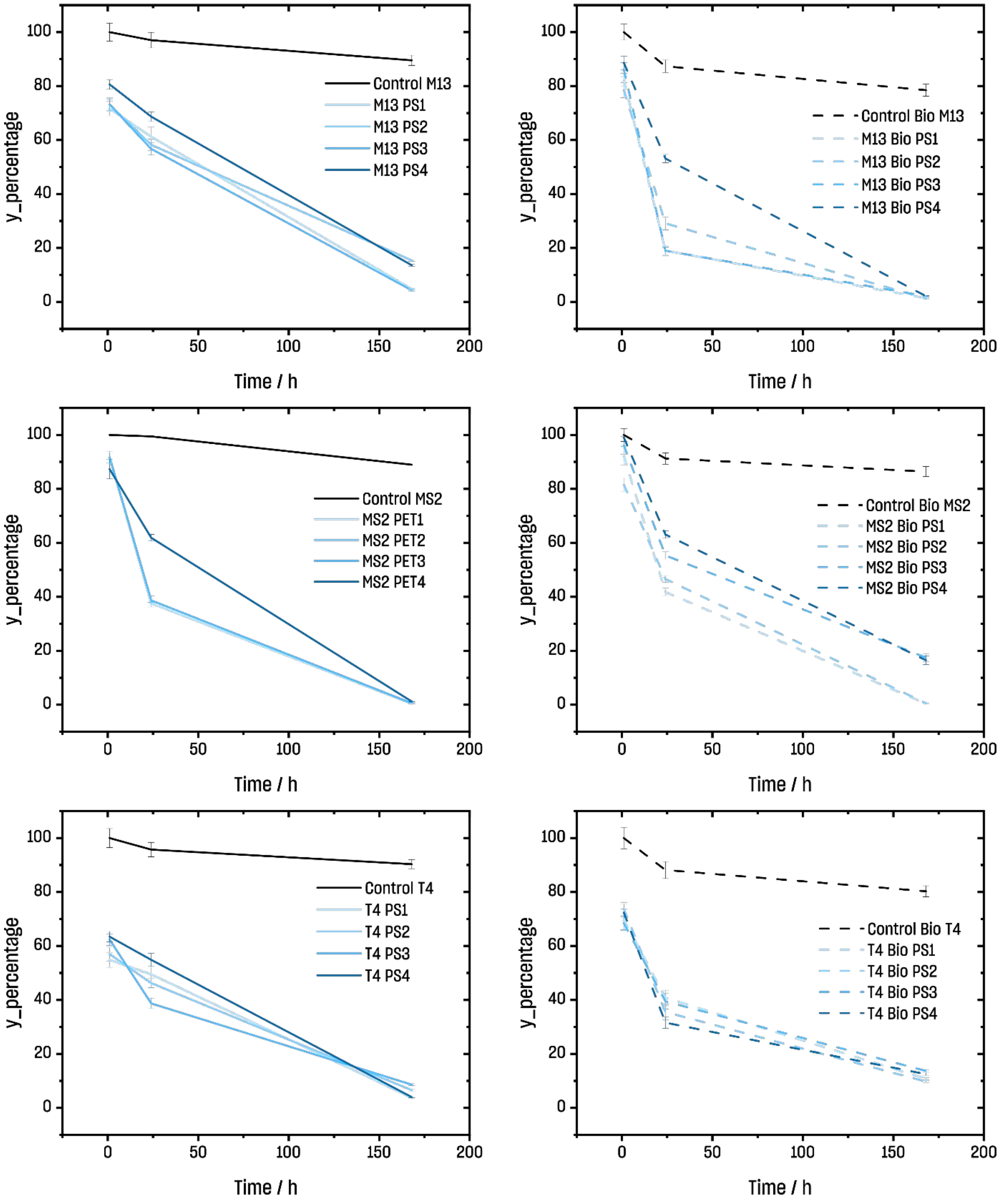

Figure S10. Experimental data for PS. Time dependence of active bacteriophages in TM buffer (left) or biomedium (right) for various polymer sizes (POLYMER1-4), as a percentage of active bacteriophages in control experiments for 1 h.

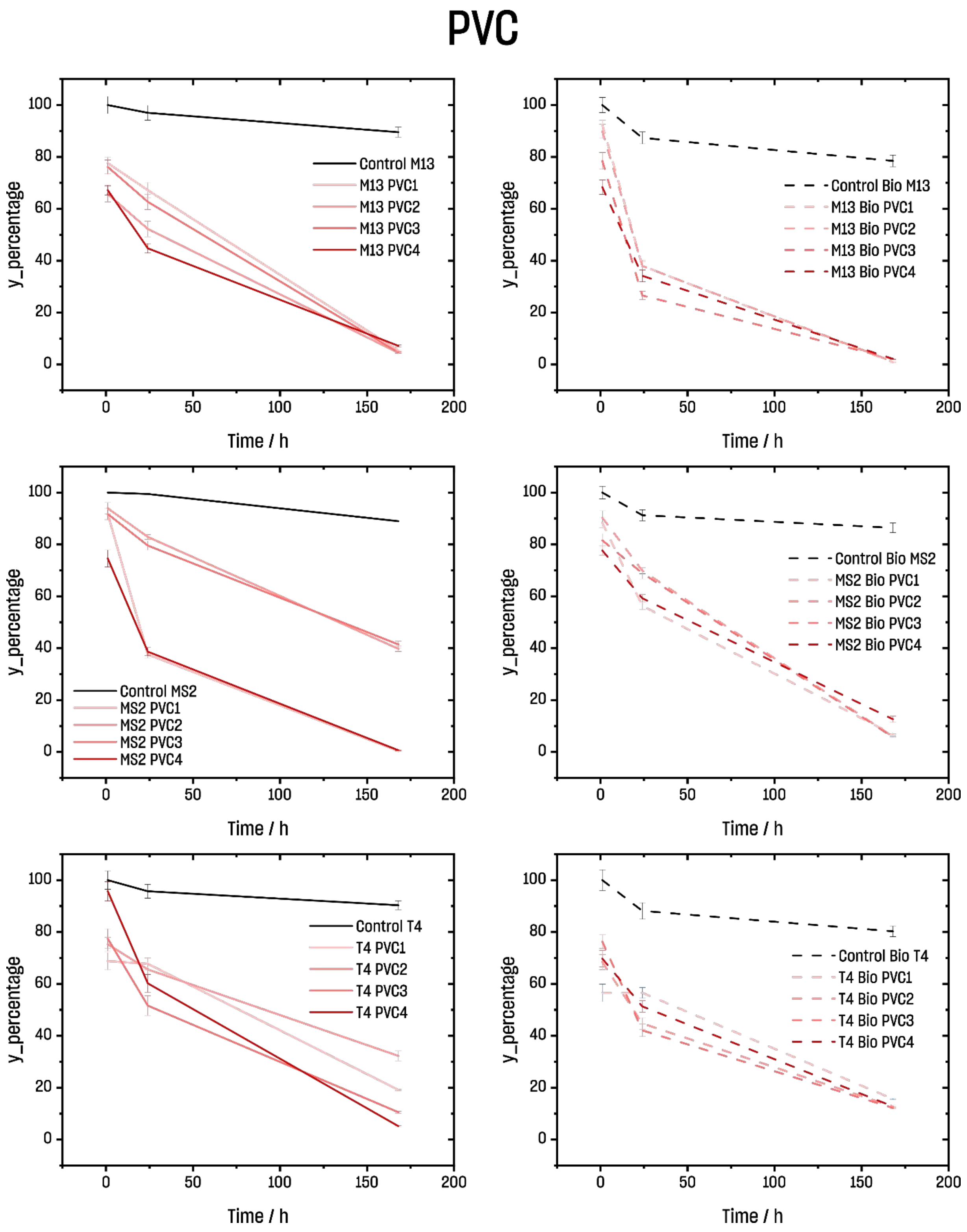

Figure S11. Experimental data for PVC. Time dependence of active bacteriophages in TM buffer (left) or biomedium (right) for various polymer sizes (POLYMER1-4), as a percentage of active bacteriophages in control experiments for 1 h.

PET

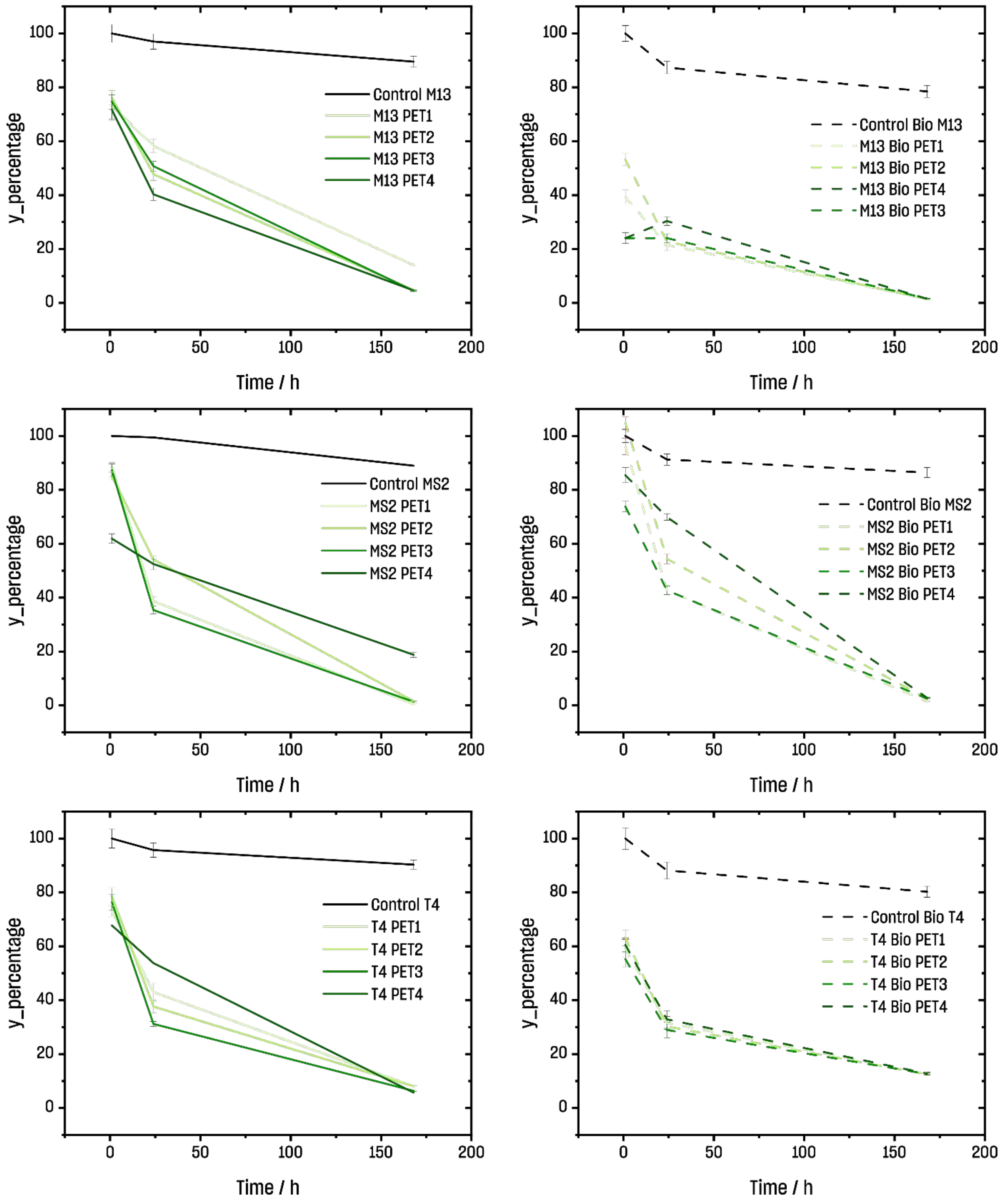

Figure S12. Experimental data for PET. Time dependence of active bacteriophages in TM buffer (left) or biomedium (right) for various polymer sizes (POLYMER1-4), as a percentage of active bacteriophages in control experiments for 1 h.

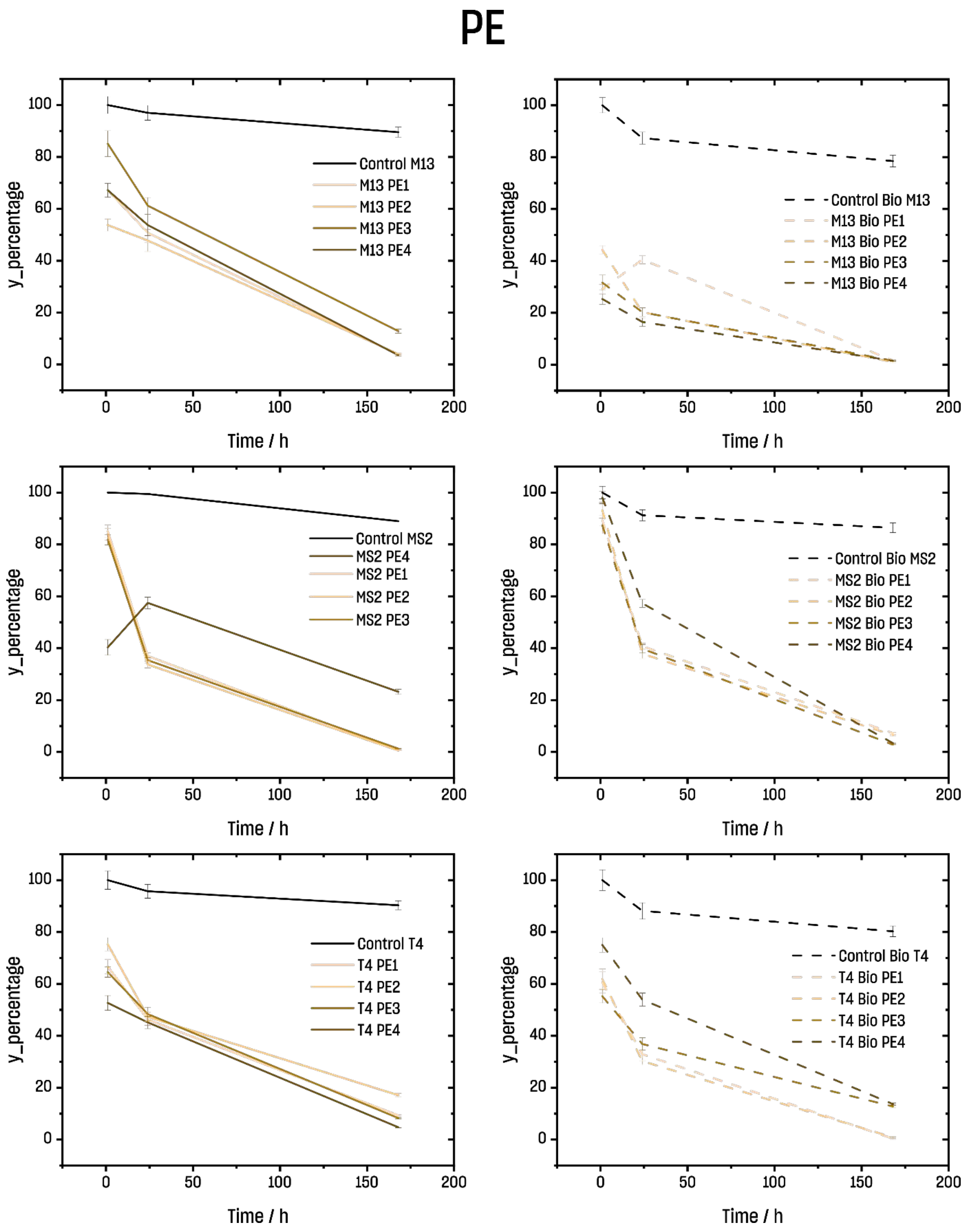

Figure S13. Experimental data for PE. Time dependence of active bacteriophages in TM buffer (left) or biomedium (right) for various polymer sizes (POLYMER1-4), as a percentage of active bacteriophages in control experiments for 1 h.

2. Multivariate linear models

In our previous paper [2], we showed that heteroaggregation of bacteriophages and microplastics occurs, which decreases the number of active phages in aquatic environments. We provided evidence that DLVO theory can explain the complex interactions driving that process. Here, we performed experiments that introduced more data to the model regarding the size of the microparticles and the presence of biomedium.

The description of heteroaggregation of biocolloids with other particles is a difficult challenge. The complexity of the systems and the abundance of possible interactions, especially in the natural environment, makes it impossible to rely on the classical DLVO theory [3]. Many more phenomena have to be considered, such as the electric double layer, temperature, mobilities of the colloids, nature of bonds, size and shape of the particles, maximal number and ratio of items within a single aggregate, and biological processes. All of those factors influence the kinetics of the heteroaggregation. To overcome these difficulties, we present an approach to the problem by calculating linear regression models. The series of experiments being a dataset to train the model, were performed to collect as much as possible of relevant physicochemical parameters. The common polymers of industrial sources were shredded and sieved, then characterized and supported by literature data. Analogically, three representatives of the most typical kinds of bacteriophages were described by experimental, physicochemical parameters. The variables and their description were attached to the database.xmlx.

Multivariate linear regression allows he description of dependent variable y with independent variables X. β parameters are found with the ordinary least squares method.The model can be further modified by introducing functions of a dependent variable and independent variables. ε is a random variable.

$$y = X\beta + \varepsilon$$

$$y = \begin{bmatrix} y_1 \\ \vdots \\ y_i \end{bmatrix}, X = \begin{bmatrix} x_{1,1} & \cdots & x_{1j} \\ \vdots & \ddots & \vdots \\ x_{i1} & \cdots & x_{ij} \end{bmatrix}, \beta = \begin{bmatrix} \beta_1 \\ \vdots \\ \beta_j \end{bmatrix}, \varepsilon = \begin{bmatrix} \varepsilon_1 \\ \vdots \\ \varepsilon_i \end{bmatrix}$$

Hence, we look for an estimation of the observed phenomena with the following equation:

$$f_y(y\_pfu) = \sum \beta_i f_i(var_i)$$

Any linear regression results based solely on physicochemical parameters did not provide reliable results. Regressions that assumed heteroaggregation as a first-order or second-order process showed massive deviations from a normal distribution of residuals of the models, and the first-order or second-order kinetical approach was not a correct construction of the model.

To overcome the difficulties, the database was divided due to time. Therefore, three subdatasets of experimental results were analyzed (for 1 h, 24 h, and 168 h). Initial estimation of the models suggested that *y_percentage* and *ln_y_percentage* were promising in the distribution of model residuals for a correct structural form.

The linear regressions were optimized with a backstep method. Regression was performed for initial variables, and the less significant variable (the highest p-value) was eliminated from the model. Then, the next regression was calculated. The procedure was repeated until all remaining variables were significant (p-value < 0.05).

However, even though some regressions reached R^2 of over 80%, the results were unreliable as the final models contained many highly correlated variables (e.g., sets of zeta-potential-related variables or related to the radius of microplastic particles based on BET measurements). Most of the models did not pass the diagnostic test; therefore, we decided that conclusions from them were too risky as they could have been misinterpreted. Eventually, we present two approaches that were used in our previous paper.

2.1. Results (Python code)

The presented results were generated with Python code provided below. The results presented below contain detailed regression results and diagnostic tests (RESET test, Breusch-Pagan test, White test, Omnibus K2 test, VIF, correlation matrices). For the convenience of the analysis with many variables, sizes 1-4 are referred to as A-D, respectively.

```
import pandas as pd
import statsmodels.api as sm
from statsmodels.stats.outliers_influence import variance_inflation_factor
import statsmodels.stats.diagnostic as smd
from statsmodels.stats.outliers_influence import reset_ramsey
import scipy.stats as stats
```

```python
import matplotlib.pyplot as plt
import datetime

current_time = datetime.datetime.now()
timestamp = current_time.strftime("%Y%m%d-%H%M%S")

# importing data

df = pd.read_csv("DATABASE_FILE", delimiter=";")
df = df.select_dtypes(include=['int64', 'float64'])

# defining dependent and independend variables

dep_var = 'DEPENDENT_VARIABLE'
indep_vars = [col for col in df.columns if col != dep_var]
indep_variables = ["SET OF INITIAL VARIABLES"]
print(indep_variables)

# Removing insignificant variables

while len(indep_variables) > 0:

 X = sm.add_constant(df[indep_variables])
 y = df[dep_var]
 model = sm.OLS(y, X).fit()
 max_p_value = max(model.pvalues)

 if max_p_value > 0.05:
 excluded_variable = model.pvalues.idxmax()
 indep_variables.remove(excluded_variable)
 print(indep_variables)
 print(excluded_variable)
 else:
 break

X = sm.add_constant(df[indep_variables])
y = df[dep_var]
model = sm.OLS(y, X).fit()

print(model.summary())

# Diagnostics of the model

vif = pd.DataFrame()
vif['VIF'] = [variance_inflation_factor(X.values, i) for i in range(X.shape[1])]
```

```python
vif['Variable'] = X.columns
average_vif = vif['VIF'].mean()

print(f"Average VIF: {average_vif:.2f}")
print(vif)

reset_test = reset_ramsey(model, degree=5)
f_value = reset_test.fvalue
p_value = reset_test.pvalue
white_test = smd.het_white(model.resid, model.model.exog)
bp_test = smd.het_breuschpagan(model.resid, model.model.exog)
omni_test = stats.jarque_bera(model.resid)

correlation_matrix = df[indep_variables].corr()
print(correlation_matrix)

# Histogram of residuals

plt.hist(model.resid, bins='auto', alpha=1, color="green", edgecolor='black', linewidth=2)
plt.gca().spines['top'].set_linewidth(2)
plt.gca().spines['right'].set_linewidth(2)
plt.gca().spines['bottom'].set_linewidth(2)
plt.gca().spines['left'].set_linewidth(2)
plt.gca().tick_params(axis='x', width=2, labelsize=15)
plt.gca().tick_params(axis='y', width=2, labelsize=15)
plt.xlabel('Residuals', fontsize=18)
plt.ylabel('Frequency', fontsize=18)
plt.savefig(f'residuals {timestamp}.svg', transparent=True)
plt.savefig(f'residuals {timestamp}.png', transparent=True)

with open('model_summary.txt', 'a') as f:
 f.write('\n' + '\n' + '\n' + '\n' + model.summary().as_text())
 f.write(f"\nTest RESET : F={str(f_value)}, p={str(p_value)}")
 f.write(
 f"\nTest White: LM={str(white_test[0])}, p-LM={str(white_test[1])}, F={str(white_test[2])},
p-F={str(white_test[3])}")
 f.write(
 f"\nTest Breusch-Pagan: LM={str(bp_test[0])}, p-LM={str(bp_test[1])}, F={str(bp_test[2])}, p-
F={str(bp_test[3])}")
 f.write(f"\nTest Omnibus K2: chi2={str(omni_test[0])}, p={str(omni_test[1])}")
 f.write(f"\n\n Variance Inflation Factor:\n" + vif.to_string())
 f.write(f"\n\n Average VIF: " + str(average_vif))
 f.write(f"\n\n Correlation matrix:\n" + correlation_matrix.to_string())
```

2.2. Coarse Models

The following regressions were calculated for binary variables describing the presence of biomedium (base: TM buffer), the presence of certain bacteriophage (base: MS2), the presence of certain polymer particles (base: PET), the size of the particles (as a categorical variable). However, interpreting the coefficients of these results separately is misleading as they are a linear transformation, and the total impact of a certain polymer may be, e.g., included in size-related variables.

Coarse models 1 h

MODEL A

```
                            OLS Regression Results
==============================================================================
Dep. Variable:        ln_y_percentage   R-squared:                       0.421
Model:                            OLS   Adj. R-squared:                  0.357
Method:                 Least Squares   F-statistic:                     6.617
Date:                Tue, 09 Jul 2024   Prob (F-statistic):           1.25e-07
Time:                        14:40:09   Log-Likelihood:                 5.5634
No. Observations:                 102   AIC:                             10.87
Df Residuals:                      91   BIC:                             39.75
Df Model:                          10
Covariance Type:            nonrobust
==============================================================================
                 coef    std err          t      P>|t|      [0.025      0.975]
------------------------------------------------------------------------------
const          4.8309      0.107     44.974      0.000       4.618       5.044
biomedium     -0.0891      0.048     -1.854      0.067      -0.185       0.006
m13           -0.3144      0.059     -5.343      0.000      -0.431      -0.198
t4            -0.2292      0.059     -3.896      0.000      -0.346      -0.112
ps             0.1752      0.070      2.502      0.014       0.036       0.314
pvc            0.1802      0.070      2.573      0.012       0.041       0.319
pe            -0.0537      0.070     -0.767      0.445      -0.193       0.085
size_a        -0.4194      0.119     -3.532      0.001      -0.655      -0.184
size_b        -0.3769      0.119     -3.174      0.002      -0.613      -0.141
size_c        -0.4285      0.119     -3.608      0.001      -0.664      -0.193
size_d        -0.4881      0.119     -4.111      0.000      -0.724      -0.252
==============================================================================
Omnibus:                       28.533   Durbin-Watson:                   0.843
Prob(Omnibus):                  0.000   Jarque-Bera (JB):               46.824
Skew:                          -1.219   Prob(JB):                     6.80e-11
Kurtosis:                       5.251   Cond. No.                         14.0
==============================================================================
```

MODEL B

```
                            OLS Regression Results
==============================================================================
Dep. Variable:        ln_y_percentage   R-squared:                       0.399
Model:                            OLS   Adj. R-squared:                  0.340
Method:                 Least Squares   F-statistic:                     6.790
Date:                Tue, 09 Jul 2024   Prob (F-statistic):           2.01e-07
Time:                        14:52:12   Log-Likelihood:                 3.6715
No. Observations:                 102   AIC:                             12.66
Df Residuals:                      92   BIC:                             38.91
Df Model:                           9
Covariance Type:            nonrobust
==============================================================================
                 coef    std err          t      P>|t|      [0.025      0.975]
------------------------------------------------------------------------------
const          4.7864      0.106     45.122      0.000       4.576       4.997
m13           -0.3144      0.060     -5.274      0.000      -0.433      -0.196
t4            -0.2292      0.060     -3.845      0.000      -0.348      -0.111
ps             0.1752      0.071      2.470      0.015       0.034       0.316
pvc            0.1802      0.071      2.540      0.013       0.039       0.321
pe            -0.0537      0.071     -0.757      0.451      -0.195       0.087
size_a        -0.4194      0.120     -3.486      0.001      -0.658      -0.180
size_b        -0.3769      0.120     -3.133      0.002      -0.616      -0.138
size_c        -0.4285      0.120     -3.562      0.001      -0.667      -0.190
size_d        -0.4881      0.120     -4.058      0.000      -0.727      -0.249
==============================================================================
Omnibus:                       34.164   Durbin-Watson:                   0.805
Prob(Omnibus):                  0.000   Jarque-Bera (JB):               64.297
Skew:                          -1.376   Prob(JB):                     1.09e-14
Kurtosis:                       5.750   Cond. No.                         12.9
==============================================================================
```

MODEL C

```
                            OLS Regression Results
==============================================================================
Dep. Variable:           y_percentage   R-squared:                       0.492
Model:                            OLS   Adj. R-squared:                  0.436
Method:                 Least Squares   F-statistic:                     8.807
Date:                Tue, 09 Jul 2024   Prob (F-statistic):           5.95e-10
Time:                        14:52:32   Log-Likelihood:                -404.53
No. Observations:                 102   AIC:                             831.1
Df Residuals:                      91   BIC:                             859.9
Df Model:                          10
Covariance Type:            nonrobust
==============================================================================
                 coef    std err          t      P>|t|      [0.025      0.975]
------------------------------------------------------------------------------
const        114.1493      5.986     19.069      0.000     102.258     126.040
biomedium     -3.3474      2.677     -1.250      0.214      -8.665       1.970
m13          -19.6922      3.279     -6.006      0.000     -26.205     -13.179
t4           -17.7346      3.279     -5.409      0.000     -24.248     -11.222
ps             9.7678      3.903      2.503      0.014       2.016      17.520
pvc            9.8577      3.903      2.526      0.013       2.106      17.610
pe            -3.2635      3.903     -0.836      0.405     -11.015       4.488
size_a       -30.7326      6.617     -4.644      0.000     -43.877     -17.589
size_b       -28.5820      6.617     -4.319      0.000     -41.726     -15.438
size_c       -30.9056      6.617     -4.671      0.000     -44.050     -17.762
size_d       -34.3246      6.617     -5.187      0.000     -47.469     -21.181
==============================================================================
Omnibus:                        7.585   Durbin-Watson:                   1.003
Prob(Omnibus):                  0.023   Jarque-Bera (JB):                7.123
Skew:                          -0.582   Prob(JB):                       0.0284
Kurtosis:                       3.567   Cond. No.                         14.0
==============================================================================
```

MODEL D

```
                            OLS Regression Results
==============================================================================
Dep. Variable:           y_percentage   R-squared:                       0.483
Model:                            OLS   Adj. R-squared:                  0.433
Method:                 Least Squares   F-statistic:                     9.554
Date:                Tue, 09 Jul 2024   Prob (F-statistic):           3.73e-10
Time:                        15:00:22   Log-Likelihood:                -405.40
No. Observations:                 102   AIC:                             830.8
Df Residuals:                      92   BIC:                             857.0
Df Model:                           9
Covariance Type:            nonrobust
==============================================================================
                 coef    std err          t      P>|t|      [0.025      0.975]
------------------------------------------------------------------------------
const        112.4756      5.852     19.218      0.000     100.852     124.099
m13          -19.6922      3.289     -5.988      0.000     -26.224     -13.160
t4           -17.7346      3.289     -5.392      0.000     -24.267     -11.203
ps             9.7678      3.914      2.495      0.014       1.993      17.542
pvc            9.8577      3.914      2.518      0.014       2.083      17.632
pe            -3.2635      3.914     -0.834      0.407     -11.038       4.511
size_a       -30.7326      6.637     -4.630      0.000     -43.915     -17.550
size_b       -28.5820      6.637     -4.306      0.000     -41.764     -15.400
size_c       -30.9056      6.637     -4.656      0.000     -44.088     -17.723
size_d       -34.3246      6.637     -5.171      0.000     -47.507     -21.142
==============================================================================
Omnibus:                        9.614   Durbin-Watson:                   0.981
Prob(Omnibus):                  0.008   Jarque-Bera (JB):                9.499
Skew:                          -0.675   Prob(JB):                      0.00865
Kurtosis:                       3.644   Cond. No.                         12.9
==============================================================================
```

Figure S14. Linear regression results for coarse models for experimental results at 1 h.

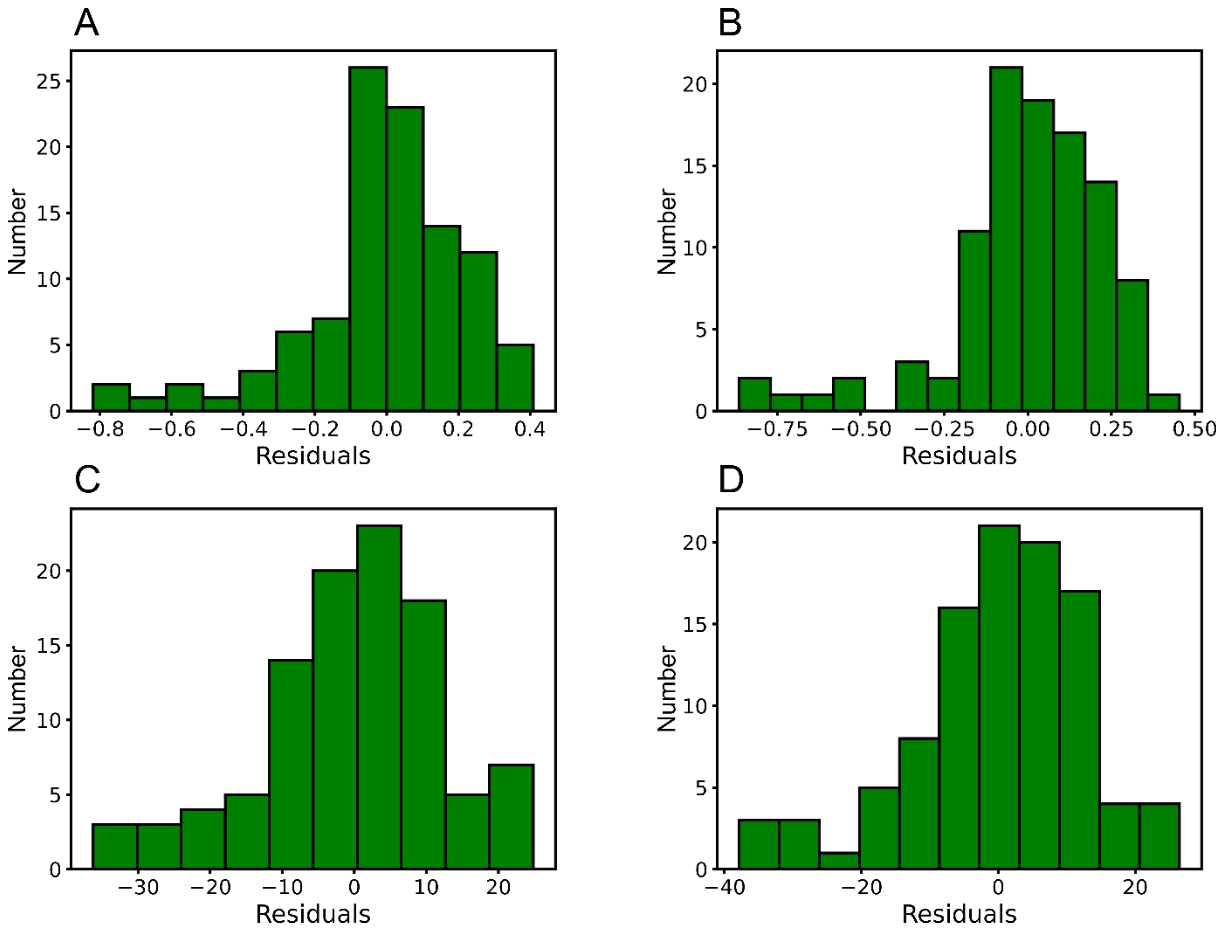

Figure S15. Residuals of the coarse models for 1 h. A-D corresponds to the models.

MODEL A

```
                            OLS Regression Results
==============================================================================
Dep. Variable:        ln_y_percentage   R-squared:                       0.547
Model:                            OLS   Adj. R-squared:                  0.497
Method:                 Least Squares   F-statistic:                     10.98
Date:                Wed, 10 Jul 2024   Prob (F-statistic):           4.87e-12
Time:                        16:28:46   Log-Likelihood:                -3.9801
No. Observations:                 102   AIC:                             29.96
Df Residuals:                      91   BIC:                             58.83
Df Model:                          10
Covariance Type:            nonrobust
==============================================================================
                 coef    std err          t      P>|t|      [0.025      0.975]
------------------------------------------------------------------------------
const          4.7863      0.118     40.579      0.000       4.552       5.021
biomedium     -0.2676      0.053     -5.072      0.000      -0.372      -0.163
m13           -0.2314      0.065     -3.582      0.001      -0.360      -0.103
t4            -0.1270      0.065     -1.966      0.052      -0.255       0.001
ps            -0.1979      0.077     -2.574      0.012      -0.351      -0.045
pet           -0.3008      0.077     -3.912      0.000      -0.454      -0.148
pe            -0.2881      0.077     -3.747      0.000      -0.441      -0.135
size_a        -0.5855      0.130     -4.491      0.000      -0.844      -0.326
size_b        -0.5910      0.130     -4.533      0.000      -0.850      -0.332
size_c        -0.6370      0.130     -4.886      0.000      -0.896      -0.378
size_d        -0.4816      0.130     -3.694      0.000      -0.741      -0.223
==============================================================================
Omnibus:                        2.829   Durbin-Watson:                   1.344
Prob(Omnibus):                  0.243   Jarque-Bera (JB):                2.750
Skew:                          -0.395   Prob(JB):                        0.253
Kurtosis:                       2.850   Cond. No.                         14.0
==============================================================================
```

MODEL B

```
                            OLS Regression Results
==============================================================================
Dep. Variable:        ln_y_percentage   R-squared:                       0.419
Model:                            OLS   Adj. R-squared:                  0.362
Method:                 Least Squares   F-statistic:                     7.362
Date:                Wed, 10 Jul 2024   Prob (F-statistic):           5.15e-08
Time:                        16:30:03   Log-Likelihood:                -16.679
No. Observations:                 102   AIC:                             53.36
Df Residuals:                      92   BIC:                             79.61
Df Model:                           9
Covariance Type:            nonrobust
==============================================================================
                 coef    std err          t      P>|t|      [0.025      0.975]
------------------------------------------------------------------------------
const          4.6525      0.129     35.928      0.000       4.395       4.910
m13           -0.2314      0.073     -3.180      0.002      -0.376      -0.087
t4            -0.1270      0.073     -1.746      0.084      -0.272       0.017
ps            -0.1979      0.087     -2.285      0.025      -0.370      -0.026
pet           -0.3008      0.087     -3.473      0.001      -0.473      -0.129
pe            -0.2881      0.087     -3.327      0.001      -0.460      -0.116
size_a        -0.5855      0.147     -3.987      0.000      -0.877      -0.294
size_b        -0.5910      0.147     -4.024      0.000      -0.883      -0.299
size_c        -0.6370      0.147     -4.337      0.000      -0.929      -0.345
size_d        -0.4816      0.147     -3.280      0.001      -0.773      -0.190
==============================================================================
Omnibus:                        2.196   Durbin-Watson:                   1.039
Prob(Omnibus):                  0.334   Jarque-Bera (JB):                1.946
Skew:                          -0.338   Prob(JB):                        0.378
Kurtosis:                       2.993   Cond. No.                         12.9
==============================================================================
```

MODEL C

```
                            OLS Regression Results
==============================================================================
Dep. Variable:           y_percentage   R-squared:                       0.644
Model:                            OLS   Adj. R-squared:                  0.605
Method:                 Least Squares   F-statistic:                     16.48
Date:                Wed, 10 Jul 2024   Prob (F-statistic):           1.51e-16
Time:                        16:30:24   Log-Likelihood:                -384.97
No. Observations:                 102   AIC:                             791.9
Df Residuals:                      91   BIC:                             820.8
Df Model:                          10
Covariance Type:            nonrobust
==============================================================================
                 coef    std err          t      P>|t|      [0.025      0.975]
------------------------------------------------------------------------------
const        103.3983      4.942     20.924      0.000      93.583     113.214
biomedium    -10.6586      2.210     -4.823      0.000     -15.048      -6.269
m13           -8.4179      2.707     -3.110      0.002     -13.794      -3.042
t4            -6.3273      2.707     -2.338      0.022     -11.704      -0.951
ps            -9.1716      3.221     -2.847      0.005     -15.571      -2.773
pet          -13.7483      3.221     -4.268      0.000     -20.147      -7.349
pe           -13.0894      3.221     -4.063      0.000     -19.488      -6.690
size_a       -39.7425      5.462     -7.276      0.000     -50.593     -28.892
size_b       -39.4936      5.462     -7.230      0.000     -50.344     -28.643
size_c       -41.0589      5.462     -7.517      0.000     -51.909     -30.209
size_d       -34.7612      5.462     -6.364      0.000     -45.611     -23.911
==============================================================================
Omnibus:                        0.217   Durbin-Watson:                   1.439
Prob(Omnibus):                  0.897   Jarque-Bera (JB):                0.395
Skew:                          -0.051   Prob(JB):                        0.821
Kurtosis:                       2.713   Cond. No.                         14.0
==============================================================================
```

MODEL D

```
                            OLS Regression Results
==============================================================================
Dep. Variable:           y_percentage   R-squared:                       0.553
Model:                            OLS   Adj. R-squared:                  0.510
Method:                 Least Squares   F-statistic:                     12.66
Date:                Wed, 10 Jul 2024   Prob (F-statistic):           7.15e-13
Time:                        16:30:34   Log-Likelihood:                -396.58
No. Observations:                 102   AIC:                             813.2
Df Residuals:                      92   BIC:                             839.4
Df Model:                           9
Covariance Type:            nonrobust
==============================================================================
                 coef    std err          t      P>|t|      [0.025      0.975]
------------------------------------------------------------------------------
const         98.0689      5.368     18.271      0.000      87.408     108.729
m13           -8.4179      3.016     -2.791      0.006     -14.409      -2.427
t4            -6.3273      3.016     -2.098      0.039     -12.318      -0.337
ps            -9.1716      3.590     -2.555      0.012     -16.302      -2.041
pet          -13.7483      3.590     -3.829      0.000     -20.879      -6.618
pe           -13.0894      3.590     -3.646      0.000     -20.220      -5.959
size_a       -39.7425      6.087     -6.529      0.000     -51.833     -27.652
size_b       -39.4936      6.087     -6.488      0.000     -51.584     -27.403
size_c       -41.0589      6.087     -6.745      0.000     -53.149     -28.969
size_d       -34.7612      6.087     -5.710      0.000     -46.851     -22.671
==============================================================================
Omnibus:                        2.855   Durbin-Watson:                   1.143
Prob(Omnibus):                  0.240   Jarque-Bera (JB):                1.910
Skew:                           0.116   Prob(JB):                        0.385
Kurtosis:                       2.371   Cond. No.                         12.9
==============================================================================
```

Figure S16. Linear regression results for coarse models for experimental results at 24 h.

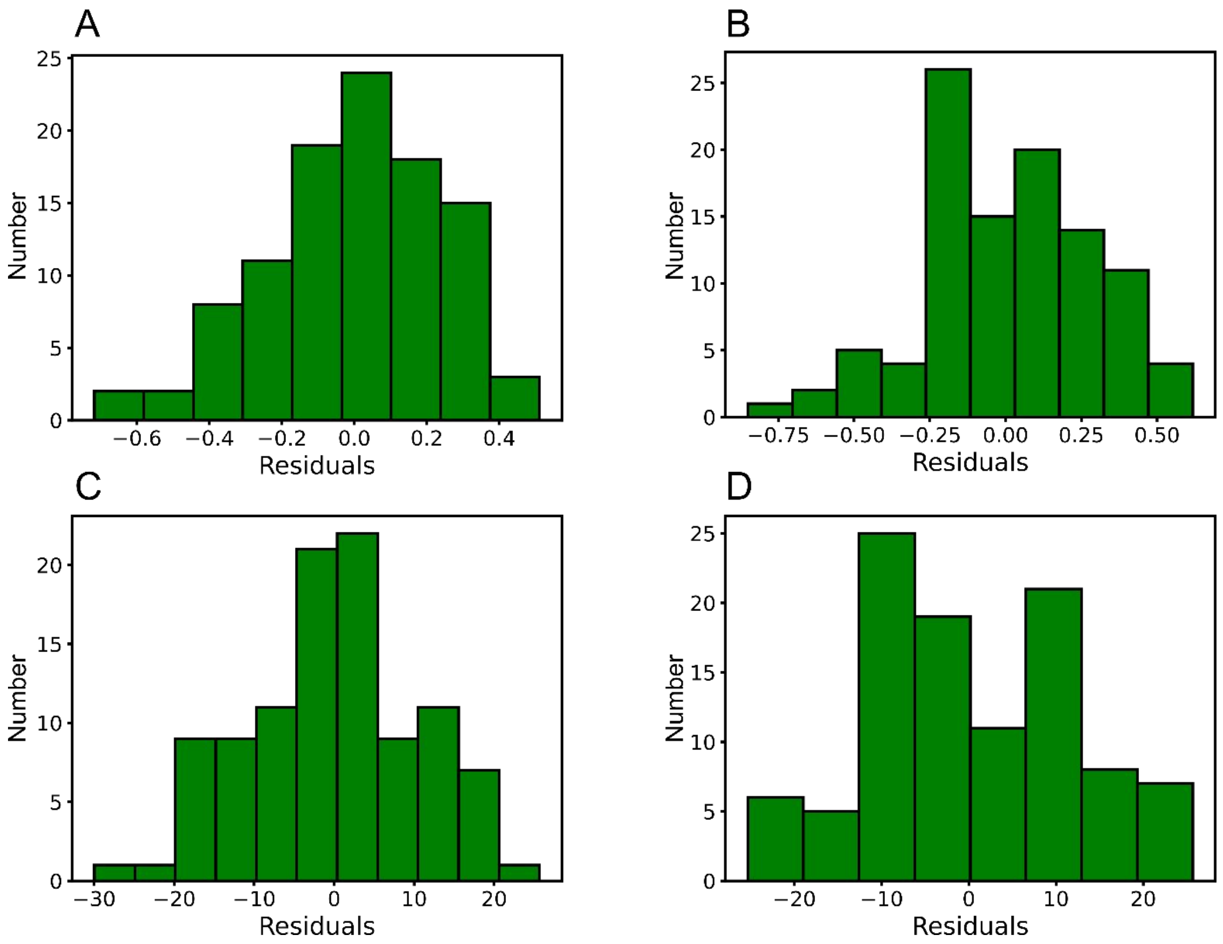

Figure S17. Residuals of the coarse models for 24 h. A-D correspond to the models.

Coarse models 168 h

MODEL A

```
                            OLS Regression Results
==============================================================================
Dep. Variable:       ln_y_percentage   R-squared:                       0.482
Model:                           OLS   Adj. R-squared:                  0.425
Method:                Least Squares   F-statistic:                     8.460
Date:               Wed, 10 Jul 2024   Prob (F-statistic):           1.34e-09
Time:                       16:44:35   Log-Likelihood:                -141.12
No. Observations:                102   AIC:                             304.2
Df Residuals:                     91   BIC:                             333.1
Df Model:                         10
Covariance Type:           nonrobust
==============================================================================
                 coef    std err          t      P>|t|      [0.025      0.975]
------------------------------------------------------------------------------
const          4.1656      0.453      9.206      0.000       3.267       5.064
biomedium     -0.2075      0.202     -1.026      0.308      -0.610       0.194
m13            0.1014      0.248      0.409      0.684      -0.391       0.594
t4             1.0598      0.248      4.276      0.000       0.567       1.552
ps            -0.6147      0.295     -2.084      0.040      -1.201      -0.029
pet           -0.4554      0.295     -1.544      0.126      -1.041       0.131
pe            -0.6216      0.295     -2.107      0.038      -1.208      -0.036
size_a        -2.9861      0.500     -5.970      0.000      -3.980      -1.992
size_b        -2.6799      0.500     -5.358      0.000      -3.674      -1.686
size_c        -2.4248      0.500     -4.848      0.000      -3.418      -1.431
size_d        -2.3691      0.500     -4.736      0.000      -3.363      -1.376
==============================================================================
Omnibus:                       0.156   Durbin-Watson:                   1.415
Prob(Omnibus):                 0.925   Jarque-Bera (JB):                0.312
Skew:                          0.068   Prob(JB):                        0.855
Kurtosis:                      2.765   Cond. No.                         14.0
==============================================================================
```

MODEL B

```
                            OLS Regression Results
==============================================================================
Dep. Variable:       ln_y_percentage   R-squared:                       0.476
Model:                           OLS   Adj. R-squared:                  0.424
Method:                Least Squares   F-statistic:                     9.277
Date:               Wed, 10 Jul 2024   Prob (F-statistic):           6.78e-10
Time:                       16:44:57   Log-Likelihood:                -141.71
No. Observations:                102   AIC:                             303.4
Df Residuals:                     92   BIC:                             329.7
Df Model:                          9
Covariance Type:           nonrobust
==============================================================================
                 coef    std err          t      P>|t|      [0.025      0.975]
------------------------------------------------------------------------------
const          4.0619      0.441      9.207      0.000       3.186       4.938
m13            0.1014      0.248      0.409      0.684      -0.391       0.594
t4             1.0598      0.248      4.275      0.000       0.567       1.552
ps            -0.6147      0.295     -2.083      0.040      -1.201      -0.029
pet           -0.4554      0.295     -1.543      0.126      -1.041       0.131
pe            -0.6216      0.295     -2.107      0.038      -1.208      -0.036
size_a        -2.9861      0.500     -5.968      0.000      -3.980      -1.992
size_b        -2.6799      0.500     -5.356      0.000      -3.674      -1.686
size_c        -2.4248      0.500     -4.846      0.000      -3.418      -1.431
size_d        -2.3691      0.500     -4.735      0.000      -3.363      -1.375
==============================================================================
Omnibus:                       0.287   Durbin-Watson:                   1.395
Prob(Omnibus):                 0.866   Jarque-Bera (JB):                0.346
Skew:                          0.122   Prob(JB):                        0.841
Kurtosis:                      2.854   Cond. No.                         12.9
==============================================================================
```

MODEL C

```
                            OLS Regression Results
==============================================================================
Dep. Variable:          y_percentage   R-squared:                       0.886
Model:                           OLS   Adj. R-squared:                  0.873
Method:                Least Squares   F-statistic:                     70.67
Date:               Wed, 10 Jul 2024   Prob (F-statistic):           1.75e-38
Time:                       16:45:09   Log-Likelihood:                -339.32
No. Observations:                102   AIC:                             700.6
Df Residuals:                     91   BIC:                             729.5
Df Model:                         10
Covariance Type:           nonrobust
==============================================================================
                 coef    std err          t      P>|t|      [0.025      0.975]
------------------------------------------------------------------------------
const         86.8804      3.159     27.506      0.000      80.606      93.154
biomedium     -2.5011      1.413     -1.771      0.080      -5.307       0.305
m13           -2.9203      1.730     -1.688      0.095      -6.357       0.516
t4             3.0194      1.730      1.745      0.084      -0.417       6.456
ps            -4.5913      2.059     -2.230      0.028      -8.682      -0.501
pet           -4.9169      2.059     -2.388      0.019      -9.007      -0.827
pe            -4.9923      2.059     -2.424      0.017      -9.082      -0.902
size_a       -76.5422      3.491    -21.923      0.000     -83.478     -69.607
size_b       -74.0690      3.491    -21.214      0.000     -81.004     -67.134
size_c       -74.0377      3.491    -21.206      0.000     -80.973     -67.102
size_d       -74.2550      3.491    -21.268      0.000     -81.190     -67.320
==============================================================================
Omnibus:                      45.274   Durbin-Watson:                   1.656
Prob(Omnibus):                 0.000   Jarque-Bera (JB):              119.071
Skew:                          1.637   Prob(JB):                     1.39e-26
Kurtosis:                      7.158   Cond. No.                         14.0
==============================================================================
```

MODEL D

```
                            OLS Regression Results
==============================================================================
Dep. Variable:          y_percentage   R-squared:                       0.882
Model:                           OLS   Adj. R-squared:                  0.870
Method:                Least Squares   F-statistic:                     76.40
Date:               Wed, 10 Jul 2024   Prob (F-statistic):           8.89e-39
Time:                       16:45:20   Log-Likelihood:                -341.04
No. Observations:                102   AIC:                             702.1
Df Residuals:                     92   BIC:                             728.3
Df Model:                          9
Covariance Type:           nonrobust
==============================================================================
                 coef    std err          t      P>|t|      [0.025      0.975]
------------------------------------------------------------------------------
const         85.6298      3.114     27.497      0.000      79.445      91.815
m13           -2.9203      1.750     -1.669      0.099      -6.396       0.555
t4             3.0194      1.750      1.725      0.088      -0.456       6.495
ps            -4.5913      2.083     -2.204      0.030      -8.728      -0.454
pet           -4.9169      2.083     -2.361      0.020      -9.054      -0.780
pe            -4.9923      2.083     -2.397      0.019      -9.129      -0.855
size_a       -76.5422      3.532    -21.673      0.000     -83.556     -69.528
size_b       -74.0690      3.532    -20.973      0.000     -81.083     -67.055
size_c       -74.0377      3.532    -20.964      0.000     -81.052     -67.023
size_d       -74.2550      3.532    -21.025      0.000     -81.269     -67.241
==============================================================================
Omnibus:                      53.086   Durbin-Watson:                   1.599
Prob(Omnibus):                 0.000   Jarque-Bera (JB):              162.791
Skew:                          1.878   Prob(JB):                     4.47e-36
Kurtosis:                      7.919   Cond. No.                         12.9
==============================================================================
```

Figure S18. Linear regression results for coarse models for experimental results at 168 h.

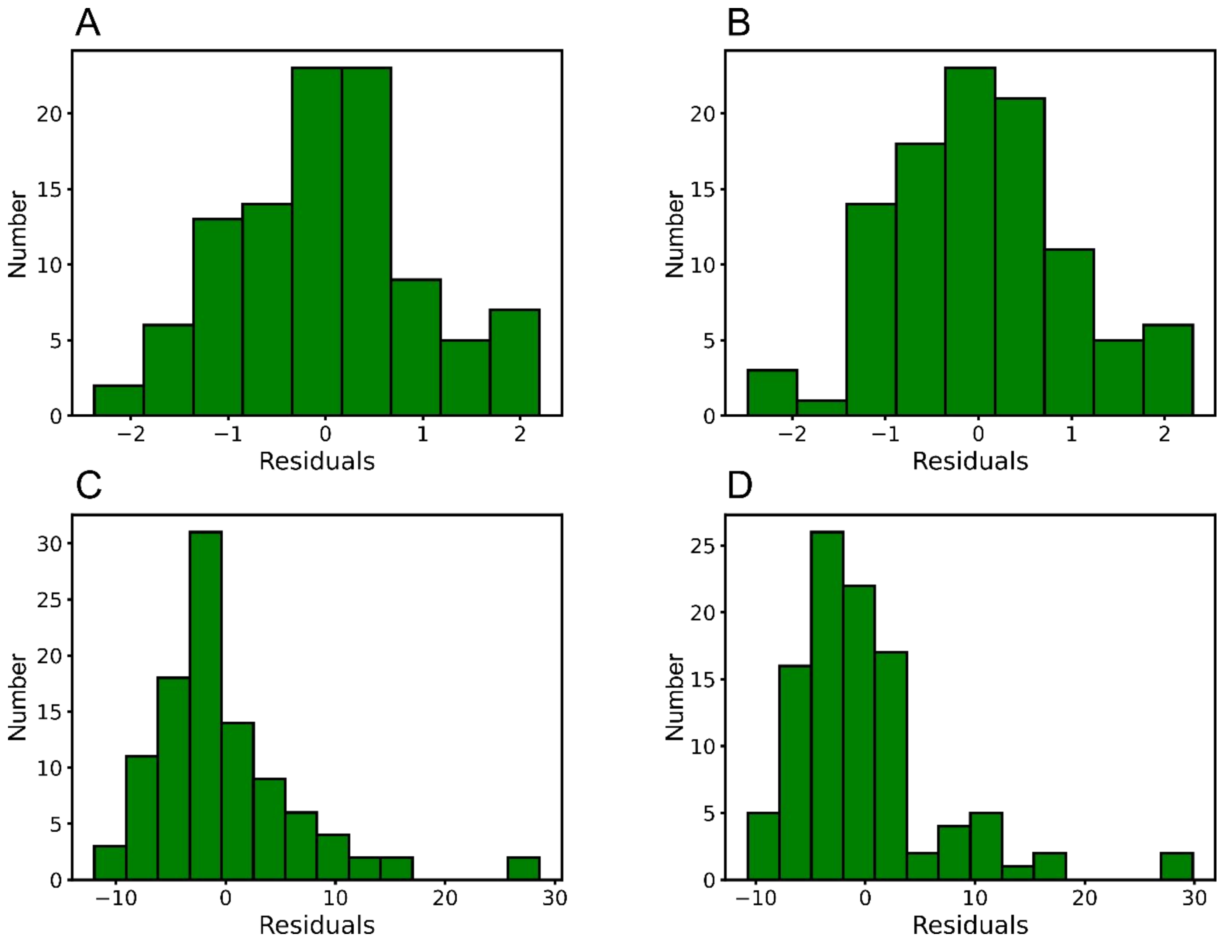

Figure S19. Residuals of the coarse models for 168 h. A-D correspond to the models.

2.3. Backstep method for interaction with type of bacteriophages

1 h

Model A

```
                            OLS Regression Results
==============================================================================
Dep. Variable:        ln_y_percentage   R-squared:                       0.477
Model:                            OLS   Adj. R-squared:                  0.450
Method:                 Least Squares   F-statistic:                     17.50
Date:                Wed, 19 Feb 2025   Prob (F-statistic):           2.78e-12
Time:                        22:55:37   Log-Likelihood:                 10.730
No. Observations:                 102   AIC:                            -9.460
Df Residuals:                      96   BIC:                             6.290
Df Model:                           5
Covariance Type:            nonrobust
===============================================================================================
                                coef    std err          t      P>|t|      [0.025      0.975]
-----------------------------------------------------------------------------------------------
const                         4.4618      0.036    125.524      0.000       4.391       4.532
int_t4_zeta_polymer           0.0114      0.003      4.330      0.000       0.006       0.017
int_t4_zeta_polymer2          0.0001   3.39e-05      3.201      0.002    4.12e-05       0.000
int_m13_zeta_polymer          0.0031      0.001      2.764      0.007       0.001       0.005
int_m13_zeta_polymer_bio      0.0270      0.003      7.708      0.000       0.020       0.034
int_m13_zeta_polymer2_bio     0.0003   4.69e-05      7.219      0.000       0.000       0.000
==============================================================================
Omnibus:                       15.720   Durbin-Watson:                   1.114
Prob(Omnibus):                  0.000   Jarque-Bera (JB):               57.843
Skew:                          -0.236   Prob(JB):                     2.75e-13
Kurtosis:                       6.659   Cond. No.                     3.70e+03
==============================================================================

Notes:
[1] Standard Errors assume that the covariance matrix of the errors is correctly specified.
[2] The condition number is large, 3.7e+03. This might indicate that there are
strong multicollinearity or other numerical problems.
Test RESET : F=21.183268838794902, p=2.0910831480912975e-12
Test White: LM=57.96278267280594, p-LM=8.785362076133282e-09, F=11.9776260703197, p-F=6.219937540853864e-13
Test Breusch-Pagan: LM=47.49718642874061, p-LM=4.498513730038447e-09, F=16.732089954209453, p-F=7.37092189144044e-12
Test Omnibus K2: chi2=57.842791541482086, p=2.7516767088307675e-13

 Variance Inflation Factor:
         VIF                   Variable
0   2.556781                      const
1  10.553446        int_t4_zeta_polymer
2   9.891242       int_t4_zeta_polymer2
3   1.956641       int_m13_zeta_polymer
4  11.056577   int_m13_zeta_polymer_bio
5  10.690756  int_m13_zeta_polymer2_bio

 Average VIF: 7.7842402325461935

 Correlation matrix:
                           int_t4_zeta_polymer  int_t4_zeta_polymer2  int_m13_zeta_polymer  int_m13_zeta_polymer_bio  int_m13_zeta_polymer2_bio
int_t4_zeta_polymer                   1.000000             -0.946178             -0.386252                 -0.250042                   0.208176
int_t4_zeta_polymer2                 -0.946178              1.000000              0.312455                  0.202269                  -0.168402
int_m13_zeta_polymer                 -0.386252              0.312455              1.000000                  0.647354                  -0.630399
int_m13_zeta_polymer_bio             -0.250042              0.202269              0.647354                  1.000000                  -0.950946
int_m13_zeta_polymer2_bio             0.208176             -0.168402             -0.630399                 -0.950946                   1.000000
```

Figure S20. Linear regression results for model A for 1 h.

Model B

```
                            OLS Regression Results
==============================================================================
Dep. Variable:           y_percentage   R-squared:                       0.492
Model:                            OLS   Adj. R-squared:                  0.466
Method:                 Least Squares   F-statistic:                     18.61
Date:                Wed, 19 Feb 2025   Prob (F-statistic):           6.91e-13
Time:                        22:56:13   Log-Likelihood:                -404.49
No. Observations:                 102   AIC:                             821.0
Df Residuals:                      96   BIC:                             836.7
Df Model:                           5
Covariance Type:            nonrobust
=============================================================================================
                              coef    std err          t      P>|t|      [0.025      0.975]
---------------------------------------------------------------------------------------------
const                      87.7032      2.083     42.103      0.000      83.568      91.838
int_t4_zeta_polymer         0.8764      0.155      5.666      0.000       0.569       1.183
int_t4_zeta_polymer2        0.0082      0.002      4.135      0.000       0.004       0.012
int_m13_zeta_polymer        0.2593      0.067      3.893      0.000       0.127       0.392
int_m13_zeta_polymer_bio    1.3993      0.205      6.826      0.000       0.992       1.806
int_m13_zeta_polymer2_bio   0.0184      0.003      6.681      0.000       0.013       0.024
==============================================================================
Omnibus:                        9.164   Durbin-Watson:                   1.216
Prob(Omnibus):                  0.010   Jarque-Bera (JB):               19.940
Skew:                          -0.138   Prob(JB):                     4.68e-05
Kurtosis:                       5.148   Cond. No.                     3.70e+03
==============================================================================

Notes:
[1] Standard Errors assume that the covariance matrix of the errors is correctly specified.
[2] The condition number is large, 3.7e+03. This might indicate that there are
strong multicollinearity or other numerical problems.
Test RESET : F=7.187774925332633, p=4.412815082657975e-05
Test White: LM=42.92396219157179, p-LM=5.131132357647274e-06, F=6.611954193847043, p-F=1.262968018359492e-07
Test Breusch-Pagan: LM=29.46643363451964, p-LM=1.877933217227713e-05, F=7.7999132557754285, p-F=3.3993712594146587e-06
Test Omnibus K2: chi2=19.93980865440789, p=4.678703949769089e-05

 Variance Inflation Factor:
         VIF                   Variable
0   2.556781                      const
1  10.553446        int_t4_zeta_polymer
2   9.891242       int_t4_zeta_polymer2
3   1.956641       int_m13_zeta_polymer
4  11.056577   int_m13_zeta_polymer_bio
5  10.690756  int_m13_zeta_polymer2_bio

 Average VIF: 7.7842402325461935

 Correlation matrix:
                           int_t4_zeta_polymer  int_t4_zeta_polymer2  int_m13_zeta_polymer  int_m13_zeta_polymer_bio  int_m13_zeta_polymer2_bio
int_t4_zeta_polymer                   1.000000             -0.946178             -0.386252                 -0.250042                   0.208176
int_t4_zeta_polymer2                 -0.946178              1.000000              0.312455                  0.202269                  -0.168402
int_m13_zeta_polymer                 -0.386252              0.312455              1.000000                  0.647354                  -0.630399
int_m13_zeta_polymer_bio             -0.250042              0.202269              0.647354                  1.000000                  -0.950946
int_m13_zeta_polymer2_bio             0.208176             -0.168402             -0.630399                 -0.950946                   1.000000
```

Figure S21. Linear regression results for model B for 1 h.

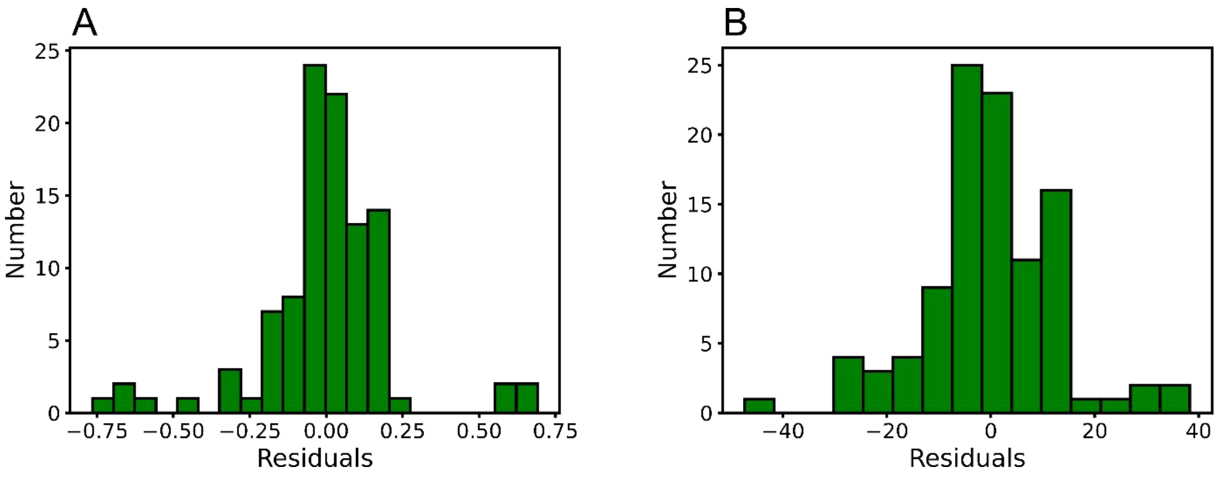

Figure S22. Residuals of the models for the ‘Backstep’ method for interaction with the type of bacteriophages for 1 h. A and B correspond to the models.

24 h

Model A

```
                            OLS Regression Results
==============================================================================
Dep. Variable:       ln_y_percentage   R-squared:                       0.705
Model:                           OLS   Adj. R-squared:                  0.676
Method:                Least Squares   F-statistic:                     24.38
Date:               Wed, 19 Feb 2025   Prob (F-statistic):           8.85e-21
Time:                       22:56:42   Log-Likelihood:                 17.845
No. Observations:                102   AIC:                            -15.69
Df Residuals:                     92   BIC:                             10.56
Df Model:                          9
Covariance Type:           nonrobust
=============================================================================================
                                coef    std err          t      P>|t|      [0.025      0.975]
---------------------------------------------------------------------------------------------
const                         4.5061      0.086     52.492      0.000       4.336       4.677
int_t4_zeta_polymer           0.0309      0.004      8.151      0.000       0.023       0.038
int_t4_zeta_polymer2          0.0003   3.87e-05      8.072      0.000       0.000       0.000
int_ms2_zeta_polymer          0.0275      0.004      7.377      0.000       0.020       0.035
int_ms2_zeta_polymer2         0.0003   3.87e-05      6.815      0.000       0.000       0.000
int_m13_zeta_polymer          0.0184      0.004      4.249      0.000       0.010       0.027
int_m13_zeta_polymer2         0.0001   4.96e-05      3.026      0.003    5.15e-05       0.000
int_t4_zeta_polymer_bio       0.0039      0.001      2.984      0.004       0.001       0.007
int_m13_zeta_polymer_bio      0.0303      0.004      6.835      0.000       0.022       0.039
int_m13_zeta_polymer2_bio     0.0003   6.19e-05      4.554      0.000       0.000       0.000
==============================================================================
Omnibus:                      11.566   Durbin-Watson:                   2.006
Prob(Omnibus):                 0.003   Jarque-Bera (JB):               13.055
Skew:                          0.659   Prob(JB):                      0.00146
Kurtosis:                      4.155   Cond. No.                     1.07e+04
==============================================================================

Notes:
[1] Standard Errors assume that the covariance matrix of the errors is correctly specified.
[2] The condition number is large, 1.07e+04. This might indicate that there are
strong multicollinearity or other numerical problems.
Test RESET : F=2.3348127654626296, p=0.061711893118078794
Test White: LM=27.13461320855441, p-LM=0.10155644139034395, F=1.5642379352717481, p-F=0.0859368899371523
Test Breusch-Pagan: LM=15.910839736429917, p-LM=0.06876699379468117, F=1.8892522476697404, p-F=0.06318293178415946
Test Omnibus K2: chi2=13.054753730522801, p=0.001462838042697037

 Variance Inflation Factor:
         VIF                   Variable
0  16.430093                      const
1  23.959057        int_t4_zeta_polymer
2  14.231895       int_t4_zeta_polymer2
3  23.237687       int_ms2_zeta_polymer
4  14.231895      int_ms2_zeta_polymer2
5  31.453492       int_m13_zeta_polymer
6  23.323177      int_m13_zeta_polymer2
7   1.721370    int_t4_zeta_polymer_bio
8  19.604977   int_m13_zeta_polymer_bio
9  20.480453  int_m13_zeta_polymer2_bio

 Average VIF: 18.86740947124362
```

Figure S23. Linear regression results for model A for 24 h.

Model B

```
                            OLS Regression Results
==============================================================================
Dep. Variable:           y_percentage   R-squared:                       0.732
Model:                            OLS   Adj. R-squared:                  0.706
Method:                 Least Squares   F-statistic:                     27.98
Date:                Wed, 19 Feb 2025   Prob (F-statistic):           1.06e-22
Time:                        22:57:04   Log-Likelihood:                -370.44
No. Observations:                 102   AIC:                             760.9
Df Residuals:                      92   BIC:                             787.1
Df Model:                           9
Covariance Type:            nonrobust
=============================================================================================
                                coef    std err          t      P>|t|      [0.025      0.975]
---------------------------------------------------------------------------------------------
const                        91.4892      3.863     23.683      0.000      83.817      99.162
int_t4_zeta_polymer           1.8760      0.171     11.003      0.000       1.537       2.215
int_t4_zeta_polymer2          0.0179      0.002     10.268      0.000       0.014       0.021
int_ms2_zeta_polymer          1.7619      0.168     10.493      0.000       1.428       2.095
int_ms2_zeta_polymer2         0.0165      0.002      9.450      0.000       0.013       0.020
int_m13_zeta_polymer          1.3150      0.195      6.731      0.000       0.927       1.703
int_m13_zeta_polymer2         0.0106      0.002      4.756      0.000       0.006       0.015
int_t4_zeta_polymer_bio       0.1776      0.059      3.001      0.003       0.060       0.295
int_m13_zeta_polymer_bio      1.0506      0.200      5.261      0.000       0.654       1.447
int_m13_zeta_polymer2_bio     0.0091      0.003      3.268      0.002       0.004       0.015
==============================================================================
Omnibus:                        4.343   Durbin-Watson:                   2.003
Prob(Omnibus):                  0.114   Jarque-Bera (JB):                3.756
Skew:                           0.452   Prob(JB):                        0.153
Kurtosis:                       3.260   Cond. No.                     1.07e+04
==============================================================================

Notes:
[1] Standard Errors assume that the covariance matrix of the errors is correctly specified.
[2] The condition number is large, 1.07e+04. This might indicate that there are
strong multicollinearity or other numerical problems.
Test RESET : F=2.5571242824328237, p=0.04421344739648863
Test White: LM=20.017139691787847, p-LM=0.3935474741209698, F=1.0537539242995992, p-F=0.4127626665685966
Test Breusch-Pagan: LM=13.31161067257301, p-LM=0.14900566676300261, F=1.5342960162279689, p-F=0.14761981425898926
Test Omnibus K2: chi2=3.755795909288609, p=0.15291119443326565

 Variance Inflation Factor:
         VIF                   Variable
0  16.430093                      const
1  23.959057        int_t4_zeta_polymer
2  14.231895       int_t4_zeta_polymer2
3  23.237687       int_ms2_zeta_polymer
4  14.231895      int_ms2_zeta_polymer2
5  31.453492       int_m13_zeta_polymer
6  23.323177      int_m13_zeta_polymer2
7   1.721370    int_t4_zeta_polymer_bio
8  19.604977   int_m13_zeta_polymer_bio
9  20.480453  int_m13_zeta_polymer2_bio

 Average VIF: 18.86740947124362
```

Figure S24. Linear regression results for model B for 24 h.

Correlation matrixes:

	int_t4_zeta_polymer	int_t4_zeta_polymer2	int_ms2_zeta_polymer	int_ms2_zeta_polymer2	int_m13_zeta_polymer	int_m13_zeta_polymer2	int_t4_zeta_polymer_bio	int_m13_zeta_polymer_bio	int_m13_zeta_polymer2_bio
int_t4_zeta_polymer	1.000000	-0.946178	-0.386252	0.312455	-0.386252	0.312455	0.647354	-0.250042	0.208176
int_t4_zeta_polymer2	-0.946178	1.000000	0.312455	-0.252758	0.312455	-0.252758	-0.612512	0.202269	-0.168402
int_ms2_zeta_polymer	-0.386252	0.312455	1.000000	-0.946178	-0.386252	0.312455	-0.250042	-0.250042	0.208176
int_ms2_zeta_polymer2	0.312455	-0.252758	-0.946178	1.000000	0.312455	-0.252758	0.202269	0.202269	-0.168402
int_m13_zeta_polymer	-0.386252	0.312455	-0.386252	0.312455	1.000000	-0.946178	-0.250042	0.647354	-0.630399
int_m13_zeta_polymer2	0.312455	-0.252758	0.312455	-0.252758	-0.946178	1.000000	0.202269	-0.612512	0.666259
int_t4_zeta_polymer_bio	0.647354	-0.612512	-0.250042	0.202269	-0.250042	0.202269	1.000000	-0.161865	0.134763
int_m13_zeta_polymer_bio	-0.250042	0.202269	-0.250042	0.202269	0.647354	-0.612512	-0.161865	1.000000	-0.950946
int_m13_zeta_polymer2_bio	0.208176	-0.168402	0.208176	-0.168402	-0.630399	0.666259	0.134763	-0.950946	1.000000

Figure S25. Correlation matrixes for variables from Models A and B for 24 h.

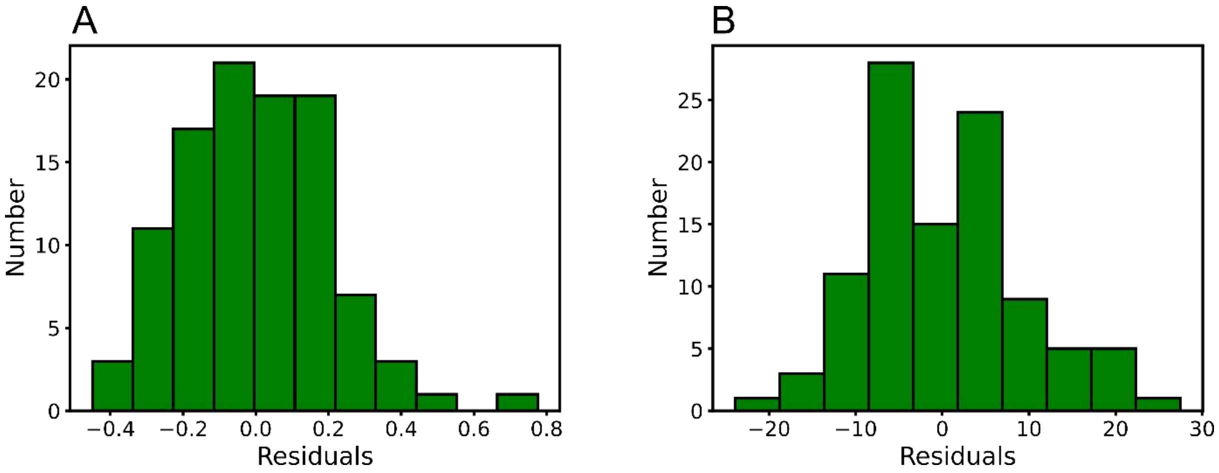

Figure S26. Residuals of the models for the 'Backstep' method for interaction with the type of bacteriophages for 24 h. A and B correspond to the models.

168 h

Model A

```
                            OLS Regression Results
==============================================================================
Dep. Variable:        ln_y_percentage   R-squared:                       0.548
Model:                            OLS   Adj. R-squared:                  0.514
Method:                 Least Squares   F-statistic:                     16.25
Date:                Wed, 19 Feb 2025   Prob (F-statistic):           7.50e-14
Time:                        22:57:52   Log-Likelihood:                -134.20
No. Observations:                 102   AIC:                             284.4
Df Residuals:                      94   BIC:                             305.4
Df Model:                           7
Covariance Type:            nonrobust
============================================================================================
                               coef    std err          t      P>|t|      [0.025      0.975]
--------------------------------------------------------------------------------------------
const                        4.4053      0.377     11.683      0.000       3.657       5.154
int_t4_zeta_polymer          0.0920      0.016      5.610      0.000       0.059       0.124
int_t4_zeta_polymer2         0.0008      0.000      4.799      0.000       0.000       0.001
int_ms2_zeta_polymer         0.1433      0.016      8.742      0.000       0.111       0.176
int_ms2_zeta_polymer2        0.0013      0.000      7.766      0.000       0.001       0.002
int_m13_zeta_polymer         0.1017      0.017      6.111      0.000       0.069       0.135
int_m13_zeta_polymer2        0.0009      0.000      5.027      0.000       0.001       0.001
int_m13_zeta_polymer_bio     0.0223      0.006      3.869      0.000       0.011       0.034
==============================================================================
Omnibus:                        5.337   Durbin-Watson:                   1.626
Prob(Omnibus):                  0.069   Jarque-Bera (JB):                7.919
Skew:                          -0.015   Prob(JB):                       0.0191
Kurtosis:                       4.365   Cond. No.                     9.38e+03
==============================================================================

Notes:
[1] Standard Errors assume that the covariance matrix of the errors is correctly specified.
[2] The condition number is large, 9.38e+03. This might indicate that there are
strong multicollinearity or other numerical problems.
Test RESET : F=1.1935741635921353, p=0.3191335071754584
Test White: LM=31.20199925017748, p-LM=0.008253886970889157, F=2.5267869215661816, p-F=0.0037912764204773323
Test Breusch-Pagan: LM=21.823512510710643, p-LM=0.0027245883800305643, F=3.6551688125717576, p-F=0.0015637255012820575
Test Omnibus K2: chi2=7.919465571680316, p=0.019068208906327315

 Variance Inflation Factor:
         VIF                  Variable
0  16.430093                     const
1  23.237687       int_t4_zeta_polymer
2  14.231895      int_t4_zeta_polymer2
3  23.237687      int_ms2_zeta_polymer
4  14.231895     int_ms2_zeta_polymer2
5  23.959057      int_m13_zeta_polymer
6  14.231895     int_m13_zeta_polymer2
7   1.721370  int_m13_zeta_polymer_bio

 Average VIF: 16.410197309364353
```

Figure S27. Linear regression results for model A for 168 h.

Model B

```
                            OLS Regression Results
==============================================================================
Dep. Variable:           y_percentage   R-squared:                       0.866
Model:                            OLS   Adj. R-squared:                  0.856
Method:                 Least Squares   F-statistic:                     87.08
Date:                Wed, 19 Feb 2025   Prob (F-statistic):           2.88e-38
Time:                        22:58:30   Log-Likelihood:                -347.37
No. Observations:                 102   AIC:                             710.7
Df Residuals:                      94   BIC:                             731.7
Df Model:                           7
Covariance Type:            nonrobust
==============================================================================================
                                coef    std err          t      P>|t|      [0.025      0.975]
----------------------------------------------------------------------------------------------
const                        83.1534      3.048     27.279      0.000      77.101      89.206
int_t4_zeta_polymer           2.6618      0.132     20.089      0.000       2.399       2.925
int_t4_zeta_polymer2          0.0217      0.001     15.781      0.000       0.019       0.024
int_ms2_zeta_polymer          2.8505      0.132     21.513      0.000       2.587       3.114
int_ms2_zeta_polymer2         0.0244      0.001     17.307      0.000       0.022       0.027
int_m13_zeta_polymer          2.7347      0.132     20.640      0.000       2.472       2.998
int_m13_zeta_polymer2         0.0210      0.001     15.290      0.000       0.018       0.024
int_ms2_zeta_polymer2_bio    -0.0013      0.001     -2.034      0.045      -0.003   -3.13e-05
==============================================================================
Omnibus:                       10.302   Durbin-Watson:                   1.494
Prob(Omnibus):                  0.006   Jarque-Bera (JB):               11.332
Skew:                           0.606   Prob(JB):                      0.00346
Kurtosis:                       4.095   Cond. No.                     1.07e+04
==============================================================================

Notes:
[1] Standard Errors assume that the covariance matrix of the errors is correctly specified.
[2] The condition number is large, 1.07e+04. This might indicate that there are
strong multicollinearity or other numerical problems.
Test RESET : F=7.034500618745881, p=5.652434827661015e-05
Test White: LM=61.29509572554824, p-LM=1.5084029384939492e-07, F=8.633485860172225, p-F=1.0729695419268936e-11
Test Breusch-Pagan: LM=56.18472037756296, p-LM=8.679326308819425e-10, F=16.467880082847433, p-F=5.4024708851844997e-14
Test Omnibus K2: chi2=11.331579123386721, p=0.0034624129045924584

 Variance Inflation Factor:
         VIF                   Variable
0  16.430093                      const
1  23.237687        int_t4_zeta_polymer
2  14.231895       int_t4_zeta_polymer2
3  23.237687       int_ms2_zeta_polymer
4  15.030134      int_ms2_zeta_polymer2
5  23.237687       int_m13_zeta_polymer
6  14.231895      int_m13_zeta_polymer2
7   1.798239  int_ms2_zeta_polymer2_bio

 Average VIF: 16.42941461471434
```

Figure S28. Linear regression results for model B for 168 h.

Correlation matrixes:

	int_t4_zeta_polymer	int_t4_zeta_polymer2	int_ms2_zeta_polymer	int_ms2_zeta_polymer2	int_m13_zeta_polymer	int_m13_zeta_polymer2	int_m13_zeta_polymer_bio
int_t4_zeta_polymer	1.000000	-0.946178	-0.386252	0.312455	-0.386252	0.312455	-0.250042
int_t4_zeta_polymer2	-0.946178	1.000000	0.312455	-0.252758	0.312455	-0.252758	0.202269
int_ms2_zeta_polymer	-0.386252	0.312455	1.000000	-0.946178	-0.386252	0.312455	-0.250042
int_ms2_zeta_polymer2	0.312455	-0.252758	-0.946178	1.000000	0.312455	-0.252758	0.202269
int_m13_zeta_polymer	-0.386252	0.312455	-0.386252	0.312455	1.000000	-0.946178	0.647354
int_m13_zeta_polymer2	0.312455	-0.252758	0.312455	-0.252758	-0.946178	1.000000	-0.612512
int_m13_zeta_polymer_bio	-0.250042	0.202269	-0.250042	0.202269	0.647354	-0.612512	1.000000

Figure S29. Correlation matrixes for variables from Models A and B for 168 h.

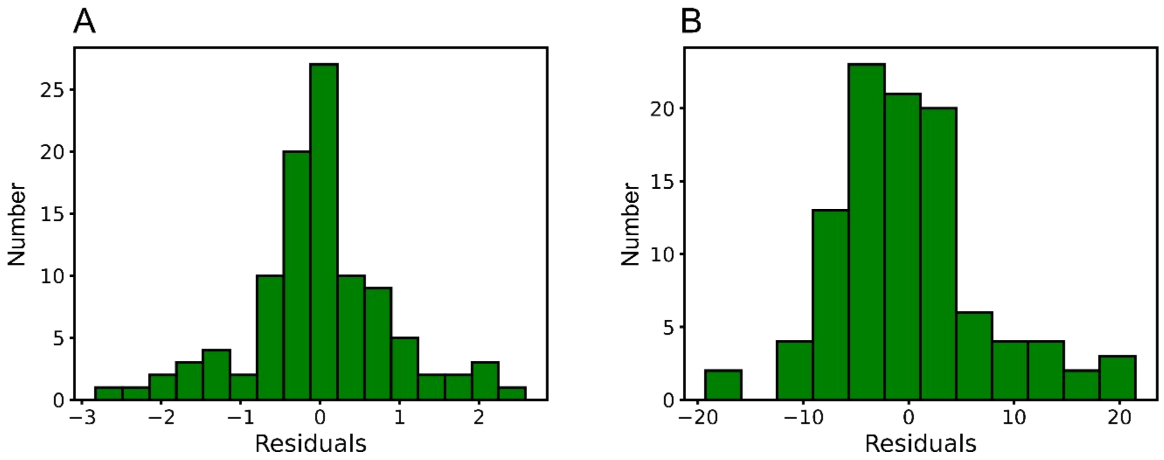

Figure S30. Residuals of the models for the Backstep method for interaction with the type of bacteriophages for 168 h. A and B correspond to the models.

Reference:

(1) Thommes, M.; Kaneko, K.; Neimark, A. V.; Olivier, J. P.; Rodriguez-Reinoso, F.; Rouquerol, J.; Sing, K. S. W. Physisorption of Gases, with Special Reference to the Evaluation of Surface Area and Pore Size Distribution (IUPAC Technical Report). *Pure Appl. Chem.* **2015**, *87* (9–10), 1051–1069. https://doi.org/10.1515/pac-2014-1117.

(2) Ochirbat, E.; Zbonikowski, R.; Sulicka, A.; Bończak, B.; Bonarowska, M.; Łoś, M.; Malinowska, E.; Hołyst, R.; Paczesny, J. Heteroaggregation of Virions and Microplastics Reduces the Number of Active Bacteriophages in Aqueous Environments. *J. Environ. Qual.* **2023**, *52* (3), 665–677. https://doi.org/10.1002/jeq2.20459.

(3) Wang, H.; Adeleye, A. S.; Huang, Y.; Li, F.; Keller, A. A. Heteroaggregation of Nanoparticles with Biocolloids and Geocolloids. *Adv. Colloid Interface Sci.* **2015**, *226*, 24–36. https://doi.org/10.1016/j.cis.2015.07.002.
